# Characterization of a Knock-In Mouse Line Expressing a Fusion Protein of κ Opioid Receptor Conjugated with tdTomato: 3-Dimensional Brain Imaging via CLARITY

**DOI:** 10.1523/ENEURO.0028-20.2020

**Published:** 2020-07-17

**Authors:** Chongguang Chen, Alex H. Willhouse, Peng Huang, Nora Ko, Yujun Wang, Bin Xu, Lan Hsuan Melody Huang, Brigitte Kieffer, Mary F. Barbe, Lee-Yuan Liu-Chen

**Affiliations:** 1 Center for Substance Abuse Research and Department of Pharmacology; 2 Cardiovascular Research Center; 3 Douglas Hospital, McGill University, Verdun, Quebec H4H 1R3, Canada; 4 Department of Anatomy and Cell Biology, Lewis Katz School of Medicine, Temple University, Philadelphia, PA 19140

**Keywords:** 3-D imaging, CLARITY, κ opioid receptor, neuroanatomy

## Abstract

Activation of κ opioid receptor (KOR) produces analgesia, antipruritic effect, sedation and dysphoria. To characterize neuroanatomy of KOR at high resolutions and circumvent issues of specificity of KOR antibodies, we generated a knock-in mouse line expressing KOR fused at the C terminus with the fluorescent protein tdTomato (KtdT). The selective KOR agonist U50,488H caused anti-scratch effect and hypolocomotion, indicating intact KOR neuronal circuitries. Clearing of brains with CLARITY revealed three-dimensional (3-D) images of distribution of KOR, and any G-protein-coupled receptors, for the first time. 3-D brain images of KtdT and immunohistochemistry (IHC) on brain sections with antibodies against tdTomato show similar distribution to that of autoradiography of [^3^H]U69,593 binding to KOR in wild-type mice. KtdT was observed in regions involved in reward and aversion, pain modulation, and neuroendocrine regulation. KOR is present in several areas with unknown roles, including the claustrum (CLA), dorsal endopiriform nucleus, paraventricular nucleus of the thalamus (PVT), lateral habenula (LHb), and substantia nigra pars reticulata (SNr), which are discussed. Prominent KtdT-containing fibers were observed to project from caudate putamen (CP) and nucleus accumbens (ACB) to substantia innominata (SI) and SNr. Double IHC revealed co-localization of KtdT with tyrosine hydroxylase (TH) in brain regions, including CP, ACB, and ventral tegmental area (VTA). KOR was visualized at the cellular level, such as co-localization with TH and agonist-induced KOR translocation into intracellular space in some VTA neurons. These mice thus represent a powerful and heretofore unparalleled tool for neuroanatomy of KOR at both the 3-D and cellular levels.

## Significance Statement

A combination of tagging κ opioid receptor (KOR) with tdTomato and tissue clearing with CLARITY enables three-dimensional (3-D) mouse brain imaging of KOR, or any G-protein-coupled receptors, for the first time. This approach reveals prominent KOR-expressing fiber bundles from caudate putamen (CP) and nucleus accumbens (ACB) to substantia nigra pars reticulata (SNr) and allows visualization of the KOR at the cellular level, including co-localization with TH and agonist-induced KOR translocation in some neurons. Regions expressing moderate to high KOR, but with no known functions, are highlighted and discussed, including claustrum (CLA), dorsal endopiriform nucleus, paraventricular nucleus of the thalamus (PVT), and lateral habenula (LHb). The mouse line will be a valuable tool for investigation of KOR neurobiology. This approach paves ways for future similar studies.

## Introduction

Activation of the κ opioid receptor (KOR) produces many effects, including analgesia, antipruritic effect, dysphoria/aversion, sedation, water diuresis and hypothermia (von Voigtlander et al., 1983; Simonin et al., 1998; Cowan et al., 2015). The selective KOR agonist nalfurafine is used in Japan for treatment of pruritus in kidney dialysis or chronic liver disease patients (Nakao and Mochizuki, 2009; Kamimura et al., 2017). In addition, KOR agonists may be useful as analgesics and water diuretics. KOR antagonists produce anxiolytic-like and antidepressant-like effects in animal models (Carlezon et al., 2009; Bruchas et al., 2010; Carr et al., 2010) and may be useful in alleviating drug craving in addicts (Shippenberg et al., 2007; Bruchas et al., 2010; Wee and Koob, 2010).

Localization of KOR protein in brains has been investigated with receptor autoradiography and immunohistochemistry (IHC). Autoradiography of binding of the selective KOR radioligand [^3^H]U69,593 or [^3^H]CI-977 has provided an excellent map of receptor distribution in the brain (Mansour et al., 1988; Slowe et al., 1999). Because of high KOR selectivity of the radioligand, receptor autoradiography has high specificity; however, the resolution is low, which does not allow visualization at the cellular level. IHC of KOR has been performed, but different KOR antibodies have yielded different results (Arvidsson et al., 1995; Drake et al., 1996; Mansour et al., 1996; Appleyard et al., 1997). Two KOR antibodies (KT2 and KOR1; Arvidsson et al., 1995; Drake et al., 1996) are more widely used and thus discussed here. KT2 and KOR1 were raised against the rat KOR 371–380 and 366–380 peptides, respectively. As discussed by Drake et al. (1996), the two antibodies revealed similarities and significant differences in KOR distribution and staining intensity. Both antibodies labeled several brain regions that have high [^3^H]U69,593 binding. However, neither labeled claustrum (CLA), which has the highest [^3^H]U69,593 binding in the brain (Unterwald et al., 1991; Wang et al., 2011). On the other hand, the striatum contained high KOR1, but low KT2 immunoreactivity. At the ultrastructural level, KOR1-immunoreactivity (IR) was localized to cell bodies and dendrites, whereas KT2-IR was found mostly in axons. The specificity of KOR antibodies from commercial sources were not adequately validated, like most commercially available antibodies against other G-protein-coupled receptors (Michel et al., 2009).

Conventional neuroanatomy methods for receptor localization, such as receptor autoradiography and IHC, are performed on brain sections, thus producing two-dimensional images. Advancements in tissue clearing with CLARITY render tissue optically transparent, yet retain tissue integrity, thus allowing three-dimensional (3-D) imaging (Chung and Deisseroth, 2013; Chung et al., 2013; Tomer et al., 2014). CLARITY involves perfusion of animals with fixatives and acrylamide-based hydrogel, cross-linking of hydrogel with proteins and nucleic acids and removal of lipid by detergents. CLARITY-cleared tissues can be imaged with or without further processing.

To generate 3-D KOR distribution in brain and to circumvent the low resolution of receptor autoradiography and the problems associated with KOR antibodies, we generated a knock-in mouse line expressing the KOR fused at the C terminus with tdTomato (tdT; KtdT). Similar approaches have been used to generate knock-in mouse lines expressing the δ opioid receptor or nociceptin/orphanin FQ receptor fused with enhanced green fluorescent protein (Scherrer et al., 2006; Ozawa et al., 2015) or the μ opioid receptor conjugated with mCherry (Erbs et al., 2015). We previously generated a mouse KtdT construct and found that when expressed in Neuro2A mouse neuroblastoma cells, mKtdT exhibited similar binding, signaling and translocation as FLAG-mKOR (Huang et al., 2013).

Here, we report generation and characterization of the KtdT mice, including behavioral responses, receptor protein and mRNA expression levels, KtdT distribution, possible co-localization with TH and agonist-induced translocation of KOR. KtdT distribution was examined with 3-D images of the KOR following clearing of brains with CLARITY, but without IHC, and IHC with antibodies against tdT on brain sections.

## Materials and Methods

### Antibodies and viral vector

Rabbit anti-red fluorescent protein (RFP) antibody was purchased from Rockland (catalog no. 600-401-379). Chicken antibodies against tyrosine hydroxylase (anti-TH) was from Abcam (ab76442). Goat anti-rabbit IgG conjugated with Alexa Fluor 594 (A11012) and goat anti-chicken IgG conjugated with Alexa Fluor 488 (A11039) were from ThermoFisher/Life Technologies. scAAV2-GFP tracer (Liu et al., 2014; titer > 10^13^ GC/ml) was a generous gift from George Smith of Temple University Lewis Katz School of Medicine.

### Materials

U50,488H and naloxone were obtained from the National Institute on Drug Abuse Drug Supply Program. [^3^H]U69,593 (60 Ci/mmol) was purchased from PerkinElmer Life Sciences. The following reagents were obtained from indicated companies: VECTASHIELD mounting media (Vector Labs), RNAeasy Mini kit (QIAGEN), Superscript II (ThermoFisher/Invitrogen), iQ SYBR green supermix (Bio-Rad), and urea (8.18 710; EMD Millipore). The following materials were purchase from Sigma-Aldrich: paraformaldehyde (PFA), compound 48/80, Kolliphor EL, phenylmethylsulfonyl fluoride, Quadrol (#122262), Triton X-100 (T8787), and Histodenz (D2158). Other commonly used chemicals were obtained from Sigma-Aldrich or ThermoFisher Scientific.

### Generation of KtdT knock-in mice

**Figure 1.**
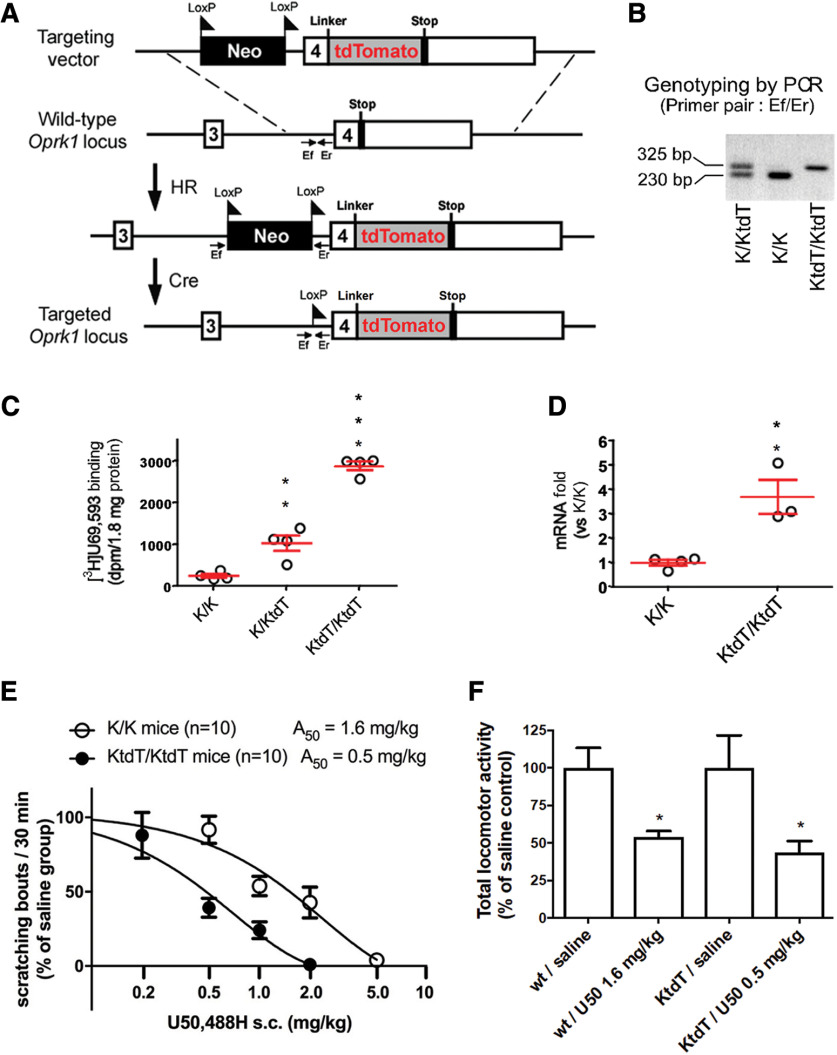
Generation and characterization of KOR-tdTomato (KtdT/KtdT) knock-in mice and comparison with the wild KOR (K/K) mice. ***A***, Targeting strategy. *Oprk1* exons 3 and 4, tdTomato cDNA, and the floxed neomycin cassette are shown as empty, gray, and black boxes, respectively. Homologous recombination (HR) was followed by Cre recombinase treatment (Cre) in ES cells. ***B***, Genotyping by PCR analysis using primer pair Ef/Er as shown in ***A*** and genomic DNA from mouse ears as templates. ***C***, [^3^H]U69,593 binding (dpm) to receptor in mouse whole-brain membranes (1.8-mg protein) was performed with 4.5 nm [^3^H]U69,593 and non-specific binding was determined by naloxone (10 μm). Data are mean ± SEM (*n* = 4) **p < 0.01 and ***p < 0.001. ***D***, Receptor mRNA levels were determined by real-time qRT-PCR with RNA preparations from mouse whole brains. Data are mean ± SEM (K/K, *n* = 4; KtdT/KtdT, *n* = 3); **p <0.01 and ***p < 0.001, compared with the wild type (K/K) by two-tailed Student’s *t* test. ***E***, U50,488H inhibited scratching behavior induced by compound 48/80 in both KtdT/KtdT and K/K mice. Male mice were pretreated subcutaneously with saline or U50,488 (0.2–5 mg/kg) and 20 min later, compound 48/80 was injected into the nape of the neck and bouts of scratching were counted for 30 min and normalized to the saline group of each mouse strain. Each value represents mean ± SEM (*n* = 10). ***F***, U50,488H inhibited novelty-induced locomotor activities in both KtdT/KtdT and K/K mice. Male mice were treated subcutaneously with saline or U50,488H (at doses of A50 values in the anti-scratching test) and put into locomotor chambers right after injections. Total locomotor activities (breaks of infrared beams) were continuously monitored using a Digiscan D. Micro System over a 90-min period. Cumulative data between 20 and 50 min postinjection are shown here. Each value represents mean ± SEM (*n* = 8). Data were analyzed by two-way ANOVA followed by Tukey’s *post hoc* test; *p < 0.05, compared with the respective saline group.

A targeting vector was constructed in which the KOR (K) gene (*oprk1*) was modified so that a floxed neomycin-resistant gene was inserted in the intron upstream of the exon four and Gly-Ser-Ile-Ala-Thr-tdTomato encoding cDNA was inserted immediately 5′ to the stop codon in the exon 4 (Fig. 1*A*). The targeting strategy was similar to those of Scherrer et al. (2006) and Ozawa et al. (2015). This construct was then transfected into embryonic stem (ES) cells. A positive ES clone with proper homologous recombination was electroporated with a Cre-expressing plasmid to excise the neomycin gene and subsequently microinjected into C57BL/6N blastocysts. The resulting animals were cross-bred with C57BL/6N mice to obtain F1 heterozygous progenies. Heterozygous mice were intercrossed to generate homologous KtdT mice (KtdT/KtdT). KtdT/KtdT mice were fertile and developed normally. Male and female homozygous (KtdT/KtdT), heterozygous (KtdT/K), or their wild-type (K/K) littermates weighing 20–23 g (8–12 weeks old) were used. Breeding was also conducted among KtdT/KtdT mice and among K/K mice. Animals were group-housed under standard laboratory conditions and kept on a 12/12 h light/dark cycle (lights on at 7 A.M.). Mice were maintained in accordance with the National Institutes of Health *Guide for the Care and Use of Laboratory Animals*. All methods used were preapproved by the Institutional Animal Care and Use Committee at the Temple University.

Mouse genotyping was conducted by PCR with total DNA isolated from mouse ears. To detect the excision of the selection marker of knock-in allele, the primer pair Ef/Er was used [5′-GATGCTGTTAATCACAGTGAGCTG −3′ (forward)/5′-CCCACAACCATAGCTCTGACAAGAG-3′ (reverse)] as diagramed in Figure 1*B*.

### Brain membrane preparation and binding of [^3^H]U69,593 to the KOR in membranes

Frozen mouse whole brains were homogenized in approximately eight volumes of 25 mm Tris-HCl buffer, pH 7.4 containing 1 mm EDTA and 0.1 mm PMSF on ice and then centrifuged at ∼100,000 × *g* for 30 min. Pellets were twice rinsed with 25 mm Tris-HCl buffer and re-suspended in 0.32 m sucrose in 50 mm Tris-HCl, pH 7.0. Suspended membranes were passed through a 26.5-G needle five times and then frozen at −80°C. KOR binding experiments were performed with [^3^H]U69,593 (∼4.5 nm) on brain membranes (1.8-mg membrane proteins). Non-specific binding was determined in the presence of naloxone (10 μm).

### Determination of KOR mRNA levels by quantitative RT-PCR (qRT-PCR)

Total RNA was isolated from mouse brains using RNAeasy Mini kit (QIAGEN). Total RNA from brain was reverse-transcribed with Superscript II reverse transcriptase (Invitrogen) and random primers. cDNA was used in PCR experiments performed with CFX Real-Time PCR system (Bio-Rad Laboratories) by using iQ SYBR green supermix (Bio-Rad). Primers for PCR were 5′-ATCACCGCTGTCTACTCTGTGG-3′ (forward) and 5′-GTGGTAGTAACCAAAGCATCTGC-3′ (reverse; https://cdn.origene.com/datasheet/mp210416.pdf), encompassing exons two and three of oprk1 gene (www.ensembl.org) and producing a 149-bp fragment from KOR cDNA. GAPDH was used as a housekeeping gene, and relative KOR mRNA levels were calculated.

### Determination of U50,488H-induced anti-scratching activities in mice

Experiments were performed according to our published procedures (Liu et al., 2019). Briefly, after habituation to observation boxes (one mouse/box) for 1 h, mice were administered subcutaneously vehicle or U50,488H at an indicated dose and 20 min later, injected with 0.1 ml of compound 48/80 (0.5 mg/ml, 50 μg, s.c.) into the nape. The number of bouts of hind leg scratching of the neck was counted for 30 min. The relative percentage of scratching was calculated as follows:

$$\frac{meannumber\;of\;scratches\;by\;treatment\;group}{meannumber\;of\;scratches\;by\;control\;group}\;\times \;100\%.$$

Typically, saline-treated mice scratched 260 ± 30 (mean ± SEM) times. A_50_ value of U50,488H was determined by plotting dose against % scratch and using linear regression to obtain a best-fit line (Prism 6.0, GraphPad Software).

### Measurement of locomotor activities

Locomotor activities were measured as described previously (Xu et al., 2013; Liu et al., 2019) using a Locomotor Activity System (Omnitech Electronics Inc.) and eight individual activity monitors. Briefly, mice were treated with saline or U50,488H (subcutaneously) at indicated doses and placed into locomotor chambers immediately. Locomotor activities were continuously monitored for 60 min, and data were recorded continuously for 5-min intervals.

### IHC

#### Tissue fixation by perfusion of mice with 4% PFA

Adult male and female KtdT/KtdT and K/K C57BL/6N mice of two to three months old were deeply anesthetized with sodium pentobarbital (7.8 mg/30 g) and perfused transcardially with 10–20 ml 0.1 m PBS (1× PBS; pH 7.4) followed by ∼100 ml 4% PFA solution in in 0.1 m phosphate buffer (1× PB; pH 7.4). Brains were dissected, postfixed in 4% PFA in 1× PB overnight, and then placed in 30% sucrose for up to 72 h for cryoprotection.

#### IHC

Brains were frozen in O.C.T. and sectioned with a cryostat (Leica CM3050S) at a thickness of 30 μm at −18°C and placed in 10 mm PBS (8.2 mm Na_2_HPO_4_, 1.8 mm KH_2_PO_4_, NaCl 134 mm, and KCl 2.7 mm; pH 7.5) plus 0.05 NaN3% for short-term storage at 4°C. Sections were rinsed with 10 mm PBS 5 × 5 min, blocked for one hour at room temperature with the blocking buffer (5% normal goat serum, 0.1 m glycine, and 0.3% Triton X-100 in 10 mm PBS). Sections were incubated with rabbit anti-RFP at 1:1000 in the staining buffer (3% BSA and 0.3% Triton X-100 in 10 mm PBS) at 4°C overnight and washed 5 × 5 min with 10 mm PBS. Sections were then incubated with Alexa Fluor 594-conjugated goat anti-rabbit IgG (1:1000) overnight at 4°C and washed 5 × 5 min with 10 mm PBS. Sections were subsequently mounted on fluorescence-free glass slides with VECTASHIELD containing DAPI and placed at 4°C for storage for up to two months. Sections were examined under a fluorescence microscope (Nikon, ECLIPSE TE300), and some were further examined under a confocal microscope (Nikon A1R).

#### Double IHC

sections were incubated with rabbit anti-RFP at 1:1000 and chicken anti-TH at 1:1000 overnight. Following washes, sections were incubated with Alexa Fluor 594-conjugated goat anti-rabbit IgG (1:1000) and Alexa Fluor 488-conjugated goat anti-chicken IgG (1:1000). After washing, sections were mounted and examined as described above.

### Autoradiography of [^3^H]U69,593 binding to KOR in coronal brain sections

The experiments were performed per our published procedures (Wang et al., 2011; Van't Veer et al., 2013). Briefly, wild-type C56BL/6 mice were killed by decapitation and brains removed and immediately frozen in isopentane on dry ice. Frozen brains were cut at 20 μm to obtain coronal sections at −18°C, which were thaw-mounted onto gelatin-subbed slides and dried in a desiccator at 4°C. Sections were incubated with ∼5 nm [^3^H]U69,593 with or without 10 μm naloxone in 50 mm Tris-HCl buffer (pH 7.4) at room temperature for 1 h. Slides were then rinsed three times with 50 mm Tris-HCl buffer at 4°C and once with deionized water and then dried with cold air. Sections were then exposed to ^3^H-sensitive phosphor screens for approximately three weeks and images on the screens were captured with a Cyclone Storage Phosphor Scanner (Packard Bioscience).

### Clearing and imaging of mouse brains with electrophoretic tissue clearing (ETC)-CLARITY method

To gain tissue transparency, while maintaining the integrity of the brain structure and reducing the time needed, we adapted the ETC method described by Kim et al. (2015).

#### Fixation and hydrogel polymerization

Mice were anesthetized and intracardiacally perfused with hydrogel solution (4% PFA, 2% acrylamide, and 0.25% VA-044 in 0.1 m PB; pH 7.4). Brains were dissected immediately, immersed in the hydrogel solution for 3 d at 4°C with mild shaking. The tissue was then transferred to a 50-ml tube containing fresh hydrogel solution and set up in a vacuum desiccator connected to a vacuum pump and a nitrogen tank through a three-way valve. Hydrogel polymerization was initiated by applying vacuum to the desiccator for 15 min. The line was then switched to nitrogen, and the tube was tightened immediately after the desiccator was filled with nitrogen gas. The polymerization was finalized after incubation of the tube for 2 h at 37°C. The fixed brains were washed and stored in PBS containing 0.1% Triton X-100 and 0.02% sodium azide at 4°C for clearing.

#### Clearing

We constructed a “core” device by assembling two electrode chambers and one sample chamber made of acrylic plastic blocks (Grainger). The sample chamber was separated from the electrode chambers by dialysis membrane (Spectra/Por, #132655) and sealed by silicon rubber gasket. The brain was enveloped and thermo-sealed in polyethylene mesh and attached to the minute handle shaft of a high torque clock. The brain was then inserted in and assembled with the sample chamber and driven at 60 min per rotation. We used common laboratory instruments mainly composed of a power supply (Bio-Rad, PowerPac HC), two refrigerated condensation traps (Savant), and two peristaltic pumps (Cole-Parmer) for buffer cooling and circulation. The running buffer (in the electrode chambers) was composed of 25 mm boric acid adjusted to pH 9.0 with lithium hydroxide and 10 mm SDS. The clearing buffer (in the sample chamber) was identical to the running buffer except containing 200 mm SDS. Electrophoretic conditions were set at constant 120 W, temperature at 15°C. With this assembly, we could fully clear a single mouse brain in 48–72 h. Some cleared brains were used for imaging directly.

#### Whole brains

The cleared brains were incubated with the refractive index (RI) matching medium Diatrizoic acid, a-Sorbitol, n-Methyl-d-Glucosamime (DSMG) (75 g diatrizoic acid, 70 g α-sorbitol, 23 g *N*-methyl-d-glucosamine, and 100 g dH_2_O, RI = 1.46) for 3 d with mild shaking in light-tight tubes. For mounting whole brains, a sample chamber was constructed in which two cover slips were spaced and sealed by a 6.5-mm silicone isolator. The optically cleared brains were positioned horizontally in the chamber filled with DSMG media. Two image stacks were taken from ventral and dorsal directions separately using a confocal microscope (Nikon A1R). The microscopic settings were as follows: A 10× objective (NA 0.45, WD 4 mm), Ch2 = 488 nm for autofluorescence (as the reference), Ch3 = 561 nm for KtdT, voxel = 1.66 × 1.66 × 5 μm^3^, depth of v-stack or d-stack = 3.5 mm at Z-step = 5 μm. Z-correction (to compensate signal reduction along Z-depth) from Z-depth 0–3.5 mm: laser power was set 10–20 for Ch3, 20–30 for Ch2 correspondingly; PMT HV was set 20–40 for Ch3, 20–40 for Ch2 correspondingly. The stacks were reduced to 25% of its original size for 3-D reconstruction using NIS-elements and Fiji (ImageJ). The rebuilt 3-D brain was presented as video clips in two views (Movies 1, 2)

Movie 1. A video clip of 3-D images of KtdT/KtdT mouse brains showing KtdT distribution. Adult KtdT/KtdT mice were perfused and brains were cleared via CLARITY. Brains were imaged without IHC from dorsal and ventral sides, and images were digitally reconstructed into 3-D images. Experiments were performed on three brains with similar results. 10.1523/ENEURO.0028-20.2020.video.1

Movie 2. A video clip of optical coronal sections of KtdT/KtdT mouse brains showing KtdT distribution. See Movie 1 legend. Coronal sections were obtained digitally and are shown as video clips. Experiments were performed on three brains with similar results. 10.1523/ENEURO.0028-20.2020.video.2

#### One-millimeter sections

Cleared brains were washed with PBS (10 mm PB, pH 7.4) 3 × 2 h and then embedded in 2% agarose. Sagittal, horizontal or coronal sections were obtained using a Vibratome (Leica VT1000P) at 1-mm thickness. IHC was performed subsequently at room temperature to facilitate antibodies permeation. Sections were incubated with rabbit anti-RFP (1:200) and chicken anti-TH (1:200) for 3 d and washed with PBS 3 × 2 h. Sections were subsequently incubated with Alexa Fluor 594-conjugated goat anti-rabbit IgG (1:200) and Alexa Fluor 488-conjugated goat anti-chicken IgG (1:200) in light-tight containers for 2 d followed by washing with PBS 3 × 2 h. All the antibodies were diluted in 5% normal goat serum, 0.3% Triton X-100, 0.1 m glycine, and 0.02% sodium azide in PBS (pH 7.4). The stained sections were RI matched in Histodenz medium (80% Histodenz in 10 mm PB buffer, pH 7.4 and 0.02% sodium azide, RI = 1.461) at room temperature overnight with mild shaking. Sections were mounted in the same RI media enclosed with 1-mm silicone isolator (JTR Press-to-Seal Silicone Isolator, 19 × 32 mm, Grace Bio-Labs, Inc) on glass slides and cover-slipped. Imaging were performed similarly as described above. Z-step was set at 5 μm, and a total of ∼200 steps were taken for each section. Multichannel images were acquired sequentially to avoid bleeding. For large images, tiles of Z-stacks were acquired and stitched using NIS-elements (Nikon).

### *In situ* hybridization (ISH)

Tissue fixation by perfusion was performed as described under IHC. Advanced Cell Diagnostics (ACD) RNAscope Technology was used for ISH. Brains were frozen in OCT and tissue was cut at 14 μm, mounted onto Superfrost Plus slides and kept at −80°C for less than three months. Tissue sections was thawed at room temperature briefly, washed with 1× PBS and subsequently processed per the ACD’s protocols. Tissue sections were then permeabilized with solutions from the pretreatment kit and incubated with protease for 30 min and hybridization probes for another 2 h at 40°C.

### Anterograde tract tracing with scAAV2-GFP tracer

Adult KtdT/KtdT mice were anesthetized with ketamine/xylazine and secured to a stereotaxic frame (Kopf) before surgery. Anesthesia was maintained with 0–1% isoflurane during surgery. After incision of skin on skull, a small hole was made in the skull with a fine drill. A 33-G injector (8IC315LISPCC, PlasticsOne) was used. scAAV2-GFP tracer (0.2 μl) was delivered into the caudate putamen (CP) and nucleus accumbens (ACB) by an infusion pump (PHD2000, Harvard Apparatus) at a rate of 0.1 μl/min. Coordinates used were those in the atlas of Paxinos and Franklin (1997): CP (AP, 1.4 mm; ML, 1.3 mm; DV, −3.8 mm) and ACB (AP, 1.4 mm; ML, 1.3 mm; DV, −4.8 mm). Three to four weeks after injection, mice were perfused and fixed as described above.

### U50,488-induced KOR translocation in the ventral tegmental area (VTA)

Adult male KtdT/KtdT mice were habituated to handling and injection by injecting (subcutaneously) with vehicle (water) once a day for 3 d. The mice were then injected subcutaneously with vehicle or U50,488H in water at 5 mg/kg and 30 min later anesthetized and perfused as described above. Frozen brains were cut at −18°C to obtain coronal sections at 30 μm. IHC was performed for both tdT and ribosomal protein S6 (S6), a cytosol protein simultaneously on floating sections containing the VTA as described above.

Quantitation of KOR translocation in VTA was performed using a method modified from Scherrer et al. (2006). Labeled brain sections were mounted in VECTASHIELD media as described above, and images were acquired with a confocal microscope (Nikon A1R) and a 60× oil objective at 0.5-μm Z-steps (voxel size = 0.21 × 0.21 × 0.5 μm^3^, 60 stacks). Measurement and calculation of KOR translocation was performed with ImageJ (Fiji version) and the workflow is detailed in Extended Data Figure 8-1. Briefly, intracellular region of interest (ROI) was defined by drawing a circle with a single pixel line around the perimeter of the S6 staining, designated as circle 2. The circle 2 was uniformly enlarged by three pixels to define total area, designated as circle 3. The nuclear ROI was defined by DAPI staining and designated as circle 1. The ROIs were drawn on three focal planes across the Z-stack of each neuron at intervals more or equal than five focal planes and registered to ROI Manager of Fiji. Background autofluorescence was corrected by the Rolling Ball (100-μm radius) algorithm of Fiji. The registered ROIs were applied to KtdT channel and the intensities of red fluorescence (KtdT) were measured for each ROI. Total receptor was defined by circle three minus circle 1 and cell surface receptor as circle three minus circle 2. The fluorescence intensity was normalized by dividing the intensity by the respective area. The results of three focal planes/neuron were averaged and counted as the value of one neuron. Approximately 50 neurons/mouse were measured, and the mean values were used for statistical analysis. Four mice were used for each of vehicle-treated and U50,488H-treated groups. Image quantitation analyses were done by an observer blinded to the treatment group.

10.1523/ENEURO.0028-20.2020.f8-1 Extended Data Figure 8-1 Workflow for quantitation of KOR translocation. A five-step procedure was applied. Identify, orthogonal view moving across Z-stack was used to select and confirm KtdT-positive neurons (see Movie 3). Tracing, S6 protein, and DAPI markers were used for unbiased and KtdT activity-independent tracing, three planes/neuron were traced due to the anisotropic nature of KtdT neurons. Register, multiplane ROIs were added in ROI manager. Measure, apply all defined ROIs to KtdT channel, obtain data of intensities and go to calculation next. Download Figure 8-1, EPS file.

Movie 3. A video clip of Z-stacks of confocal images of a VTA section co-stained for KtdT (red), TH (green) and DAPI (blue). Experiments were performed on five VTAs with similar results. 10.1523/ENEURO.0028-20.2020.video.3

## Results

### Generation and characterization of a knock-in mouse line expressing KtdT

We used homologous recombination to introduce tdT cDNA into exon four of the Oprk1 mouse gene, in frame and 5′ to the stop codon (Fig. 1*A*). Genotyping was performed with PCR using a primer pair (Ef/Er) which detected the loxP site (95 bp) introduced 5′ to the exon 4 in the KtdT allele (Fig. 1*B*).

Radioligand binding was conducted using the selective KOR agonist [^3^H]U69,593 and brain membranes (1.8-mg protein/tube) prepared from wild-type (K/K), heterozygote (K/KtdT), and homozygote (KtdT/KtdT) mice. The results revealed that [^3^H]U69,593 binding in brains was much higher in KtdT/KtdT mice than in K/K mice, with a ratio of 12:1 (Fig. 1*C*). Quantitative mRNA analysis revealed that a KOR mRNA ratio of 1:3.6 in the wild-type versus KtdT/KtdT mouse brain (Fig. 1*D*).

Effects of the selective KOR agonist U50,488H on compound 48/80-induced scratch behavior was examined. U50,488H inhibited compound 48/80-induced scratching behavior in both KtdT/KtdT and K/K mice in dose-dependent fashion. U50,488H was approximately three times more potent in KtdT/KtdT mice (A_50_ = 0.5 mg/kg) than in K/K mice (A_50_ = 1.6 mg/kg; Fig. 1*E*). In addition, at a dose of ∼ A_50_ in anti-scratch test, U50,488H caused similar levels of hypolocomotion in KtdT/KtdT mice as in K/K mice (Fig. 1*F*). These results indicate that the KtdT/KtdT knock-in mouse line expresses a fully functional tdTomato-tagged KOR and has intact neuronal circuitry for these behaviors. To our knowledge, this is the only tdTomato tagged G-protein-coupled receptor knock-in mouse reported to date. We thus used these mice to examine the distribution of KtdT in the mouse brain.

### Two approaches for examination of distribution of KtdT in the mouse brain

Distribution of KtdT in the brain was examined with two approaches. The first is 3-D images of CLARITY-cleared brains without IHC staining. The second is IHC staining of brain sections with antibodies recognizing tdT. Two approaches yielded similar results.

#### 3-D image of KtdT distribution in the mouse brain

**Figure 2.**
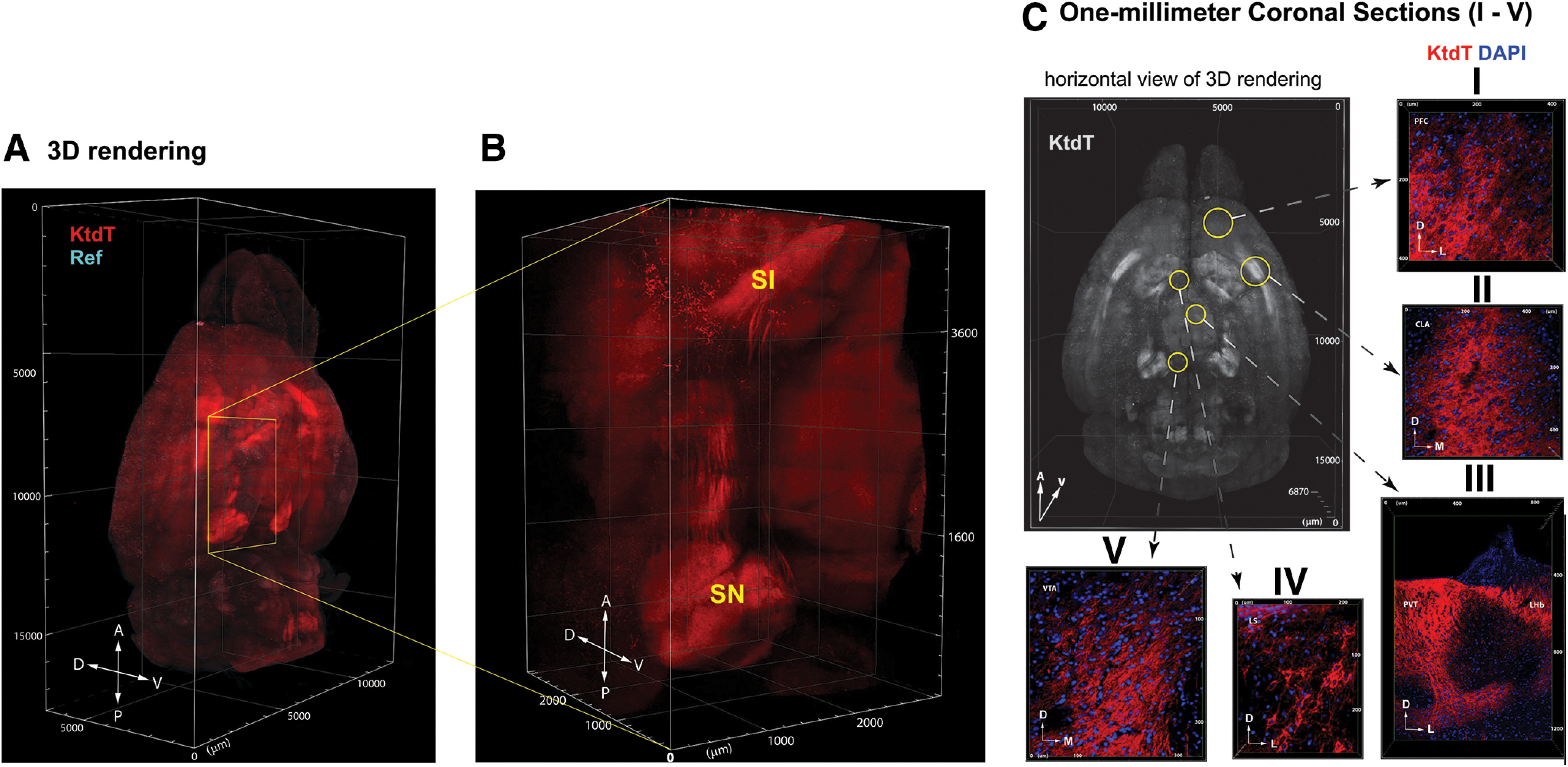
***A***, A 3-D image of mouse brain cleared with CLARITY showing brain-wide distribution of KtdT. The 3-D whole-brain image was reconstructed from ventral and dorsal stacks acquired separately with optical settings described in Materials and Methods. Directions: anterior (A), posterior (P), ventral (V), dorsal (D), lateral (L), and medial (M). The autofluorescence in green channel was used as a reference (Ref) to whole-brain volume which is pseudo-colored in cyan. Experiments were performed on three brains with similar results. See Movies 1, 2 for video clips. ***B***, An enlarged view of a portion of the 3-D image showing prominent KtdT fiber bundles between SI and SNr. ***C***, Images (I–V) were presented as optical sections from 1-mm coronal blocks of mouse brains showing KtdT neurons in the prefrontal cortex (PFC), claustrum (CLA), lateral septum (LS), ventral tagmental area (VTA), paraventricular nuleus of thalamus (PVT), lateral habenula (LHb). The brain region of the images is marked by circles in the horizontal view of 3-D rendering (gray).

Mouse brains were cleared with CLARITY. No IHC staining was performed. Cleared brains were imaged with confocal microscopy from dorsal side and ventral sides. Two sides were then reconstructed digitally and the 3-D image thus obtained is shown as a video clip in Movie 1. To the best of our knowledge, this is the first 3-D image of distribution of a G-protein-coupled receptor in brains. Figure 2*A* shows a view of a 3-D image. Figure 2*B* shows an enlarged 3-D image of a portion of the brain from the substantia innominata (SI) to SNr, both of which express high levels of KtdT. Prominent KOR-tdT-containing fibers are visible in this image. Coronal sections of the 3-D image were collected digitally and shown as video clips (Movie 2). Figure 2*C* are images obtained from 1-mm sections showing KtdT distribution at the cellular level in prefrontal cortex (PF), CLA, lateral septum (LS), VTA, paraventricular nucleus of thalamus (PVT), lateral habenula (LHb). Detailed KtdT distribution in the brain is described below.

#### IHC of KtdT showed similar distribution as that of [^3^H]U69,593 binding to the KOR in wild-type mouse brain

We also examined KtdT distribution in conventional brain sections. As sections of un-cleared KtdT/KtdT mouse brains did not yield fluorescence of sufficiently high tdT intensity, KtdT in brain sections was detected by IHC with antibodies against the RFP, which recognized tdT. Specificity of the antibodies was examined using sections containing midbrain and cerebellum of the wild-type mice and KtdT/KtdT mice. While signals were observed in the midbrain of KtdT/KtdT mice, no fluorescent signal were detected in wild-type littermates (data not shown). There was no staining in the cerebella of KtdT/KtdT mice (data not shown), which is consistent with the finding that the mouse cerebellum does not express KOR (Slowe et al., 1999). The distribution of KtdT IHC in brain sections is similar to that of KtdT in CLARITY-cleared brains without IHC (Movies 1, 2), further demonstrating the specificity of antibody.

**Figure 3.**
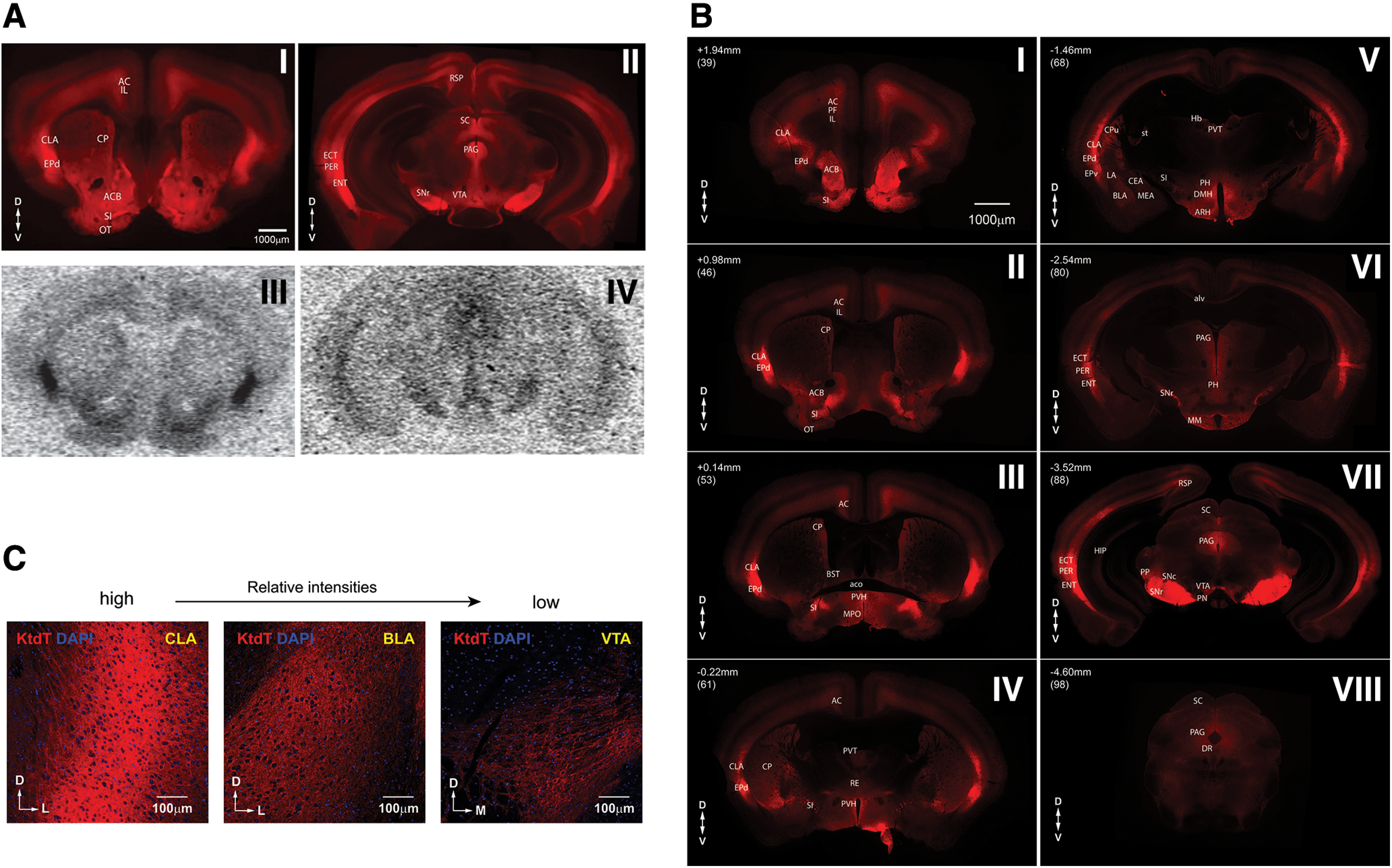
***A***, Comparison of IHC staining of KtdT (top row) with autoradiography of [^3^H]U69,593 binding to the KOR (bottom row) in two coronal brain sections (30 μm for IHC and 20 μm for autoradiography in thickness). IHC images of KtdT were captured with a wide field fluorescence microscope. Experiments were performed on three brains each with similar results. ***B***, Distribution of KtdT in coronal brain sections. IHC images of KtdT were captured with a wide field fluorescence microscope. Rostral-caudal coordinates in reference to bregma are indicated. The numbers in parenthesis are the coronal section numbers in the Mouse Atlas of Allen Brain Institute (https://mouse.brain-map.org/experiment/thumbnails/100048576?image_type=atlas). Experiments were performed on three brains each with similar results. For neuroanatomical sites, see list of abbreviations for brain nucleus and regions. ***C***, Three higher resolution images with relative intensities from high to low. The images were taken with 20× objective on confocal microscope. The regions CLA, BLA, and VTA were chosen as representatives of high to low KtdT expression levels. List of abbreviations for brain nucleus and regions (per Mouse Brain Atlas of Allen Brain Institute): A11: A11 DA neurons; aco: anterior commissure; AC: anterior cingulate cortex; ACB: nucleus accumbens; alv: alveus of hippocampus; ARH: arcuate nucleus of hypothalamus; BLA: basolateral amygdala nucleus; BMA: basomedial amygdala nucleus; BST: bed nucleus of the stria terminalis; CB: cerebellum; CEA: central amygdalar nucleus; CLA: claustrum; CP: caudate putamen; CS: superior central nucleus raphe; CTX: cortex; ECT: ectorhinal cortex; ENT: entorhinal cortex; EP: endopiriform nucleus; EPd: dorsal endopiriform nucleus; DMH: dorsomedial nucleus of the hypothalamus; DR: dorsal raphe nucleus; EW: Edinger–Westphal nucleus; Hb: habenula; HIP: hippocampus; LA: lateral amygdalar nucleus; LHb: lateral habenula; LM: lateral mammillary nucleus; MEA: medial amygdalar nucleus; MM: Medial mammary nucleus; MPO: medial preoptic area; nst: nigrostriatal tract; OB: olfactory bulb; OT: olfactory tubercle; PAG: periaqueductal gray; PER: perirhinal cortex; PF: prefrontal cortex; PH: posterior hypothalamic area; PVH: paraventricular hypothalamus nucleus; PVT: paraventricular nucleus of thalamus; RE: reuniens nucleus of thalamus; RSP: retrosplenial cortex; SI: substantia innominata; SNc: substantia nigra, pars compacta; SNr: substantia nigra, pars reticulata; st: stria terminalis; STN: subthalamic nucleus; SC: superior colliculus; VTA: ventral tegmental area.

Autoradiography of [^3^H]U69,593 binding to the KOR in wild-type mouse brains was performed for comparison with IHC of tdT signals in brains of KtdT/KtdT mice. As shown in Figure 3*A*,*B*, the distribution of KtdT immunoreactivity is consistent with that of autoradiography of [^3^H]U69,593 binding to the KOR in the wild-type mouse brain.

### Distribution of KtdT immunoreactivity in the brain

Distribution of KtdT immunoreactivity in the brain is shown in eight coronal sections, from rostral to caudal (Fig. 3*B*). The greatest signal intensity was observed in the CLA, dorsal endopiriform nucleus (EPd), ACB shell, periaqueductal gray (PAG), and SNr. High-intensity fluorescence in these regions is concordant with KOR receptor autoradiography in wild-type mice (Slowe et al., 1999). Relative intensities of KtdT in brain regions are shown in Table 1. Figure 3*C* shows higher resolution images of the CLA, basolateral amygdala (BLA) and VTA as examples for high-expressing, medium-expressing, and low-expressing brain regions, respectively.

**Table 1:** KOR distribution in brain regions of KtdT/KtdT mice

Telencephalon			Hypothalamus	
Amygdala			Arcuate nucleus	2
Central nucleus	1		Dorsomedial nucleus	3
Cortical nucleus	1		Lateral hypothalamic area	1
Basolateral nucleus	2		Mammillary nucleus	1
Medial nucleus	1		Paraventricular nucleus	2
Bed nucleus, stria terminalis	1		Periventricular area	3
Cerebral cortex			Preoptic area	1
Layer 1	1		Suprachiasmatic nucleus	2
Layer 2/3	2		Retrochiasmatic nucleus	2
Layer 4	0		Subthalamus	
Layer 5	2		SI	2
Layer 6	2		Subthalamic nucleus	2
CLA	4		Zona incerta	1
CP	1		Thalamus	
Endopiriform nucleus	4		Periventricular nucleus	2
Globus pallidus	1		Reuniens nucleus	1
Hippocampal formation				
Cornu ammonis	1		**Mesencephalon**	
Dentate gyrus	1		Edinger–Westphal nucleus	2
Septum			PAG	3
LS	1		SN	
Medial septum	1		SNc	1
SI	3		SNr	4
Ventral striatum			Superior colliculus	3
ACB core	2		Retrorubral field (A8)	3
ACB shell	3		VTA	2
Olfactory tubercle	2			
			**Pons/medulla**	
**Diencephalon**			Nucleus raphe magnus	3
Epithalamus			Parabrachial nucleus	2
LHb	1		Raphe nuclei	2
Medial habenula	1		Rostral ventral medulla	2

Number 4 indicates the highest density and 1 the lowest. Data are derived from three mice.

#### Telencephalon

KtdT signal was seen throughout the neocortex, with the notable exception of Layer IV. The most intense fluorescence in the neocortex was observed in insular and cingulate cortices, prebregma, as well as retrosplenial cortices, postbregma. The CLA as well as the EPd, which borders the ventral CLA, both exhibit high-intensity signals.

Rostral limbic and olfactory regions exhibited high fluorescence, where KtdT signal was observed in the anterior olfactory nucleus, EPd, and olfactory tubercles. The dorsomedial aspect of the dorsal striatum, which comprises the CP, exhibited modest fluorescence. Strong signal was observed throughout the ventral striatum, comprising the olfactory tubercle and ACB, with distinct, high-intensity patterns of fluorescence observed in the ACB shell; however, KtdT signal was comparatively low within the ACB core. In addition, the SI exhibited high signal intensity.

KtdT signal intensity was low within the hippocampal formation, only visible within dentate gyrus regions of the hippocampus (HIP). Signal was brighter and more intense throughout other regions of the medial temporal lobe, such as the perirhinal, ectorhinal, and entorhinal cortices. Signal of moderate intensity was observed in cortical and subcortical amygdala nuclei.

#### Diencephalon

Low-intensity signal was observed throughout the bed nucleus of the stria terminalis, adjacent to both the dorsal and rostral aspects of the olfactory and temporal limbs of the anterior commissure. KtdT signal of varying intensity was observed throughout the rostral hypothalamus, including regions within the medial preoptic area, as well as the periventricular nucleus. Postbregma hypothalamic signal was observed in arcuate nucleus, dorsomedial hypothalamic nucleus, lateral hypothalamic area, lateral mammillary nucleus, posterior hypothalamic area, ventral premammillary nucleus, and ventromedial hypothalamic nucleus.

KtdT signal was observed in the LHb, as well as in discrete thalamic regions, including PVT, and reuniens nucleus (RE), but KtdT signal appeared less widespread throughout the thalamus than in the hypothalamus. Low fluorescent intensity was observed in subthalamic regions including the lateral globus pallidus, subthalamic nucleus, ventrolateral geniculate nucleus, and zona incerta in these regions was low.

#### Mesencephalon

KtdT signal observed in the mesencephalon was brightest in the SNr and PAG. Fluorescence was also observed in the midbrain reticular formation, most notable in the VTA and dopamine (DA) cell group A8, also known as the retrorubral field in mice. Further midbrain staining was observed in the medial superior colliculus, as well as within the Edinger–Westphal and peripeduncular nuclei.

#### Pons and medulla

Staining within the lower part of the brainstem revealed striking fluorescence in several discrete raphe nuclei, including caudal linear raphe, dorsal raphe, raphe magnus, and rostral linear raphe nuclei. KtdT signal was also observed in the Barrington’s nucleus, the lateral and medial parabrachial nucleus, and the rostral ventral medulla.

### Co-staining of KtdT and TH in sagittal and horizontal sections of CLARITY-cleared brains

**Figure 4.**
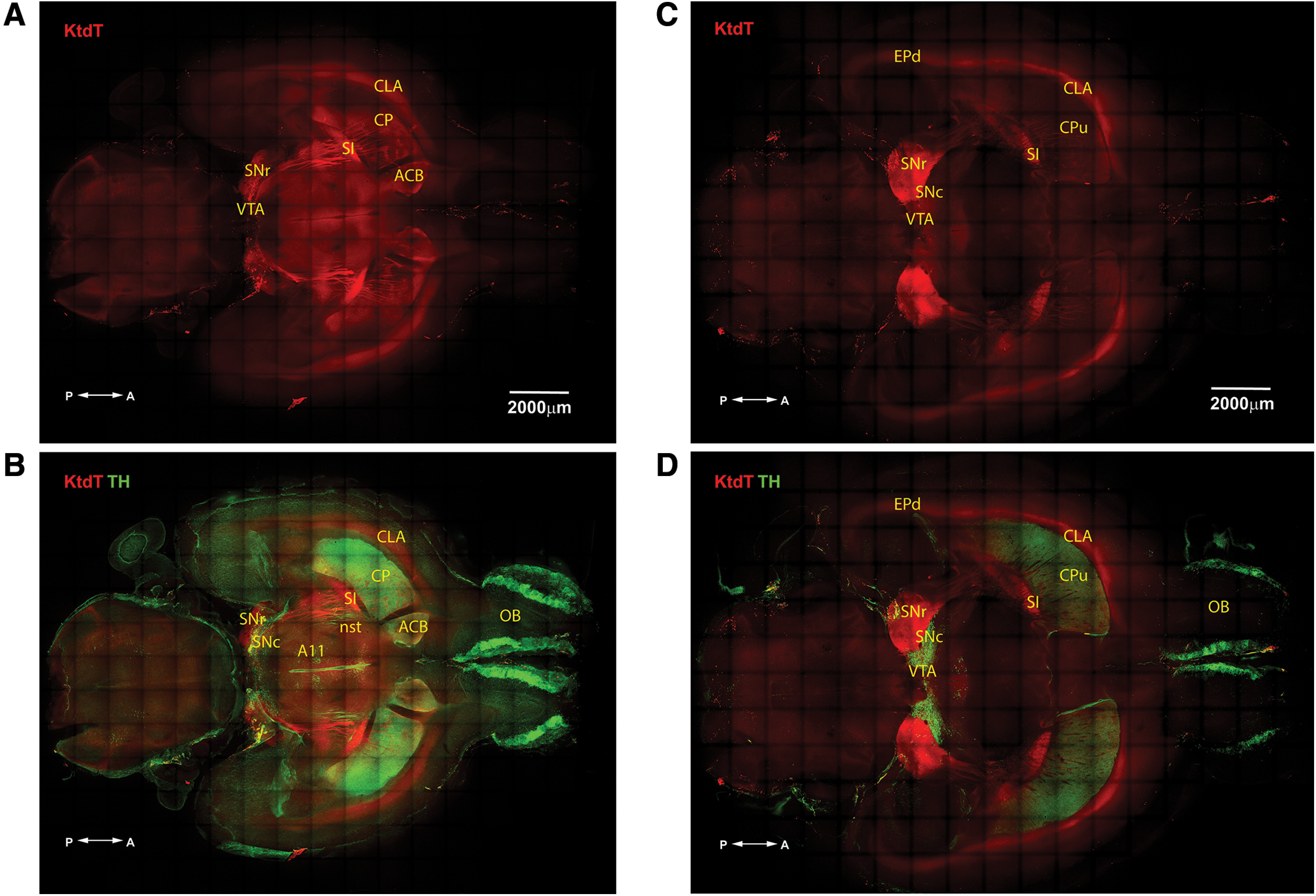
A thick horizontal brain section (1 mm) of the KtdT mouse showing IHC staining of (***A***) KtdT (red) alone and (***B***) both KtdT (red) and TH (green). KtdT/KtdT mice were perfused and cleared with the CLARITY method. Cleared brains were sectioned at 1 mm with a Vibratome, and IHC was performed on floating sections for TH and KtdT. IHC image Z-stacks were captured with a confocal microscope, and the tiles were stitched in real time with NIS-Element software. The images shown are maximum intensity projection (MaxIP) of the stacks to demonstrate long-range projections. In these images, TH-containing or KtdT-containing fibers are clearly visible between SN and CP. ***A***, ***B***, This section is approximately −4.4 mm ventral to bregma. ***C***, ***D***, This section is approximately −5.6 mm ventral to bregma.

**Figure 5.**
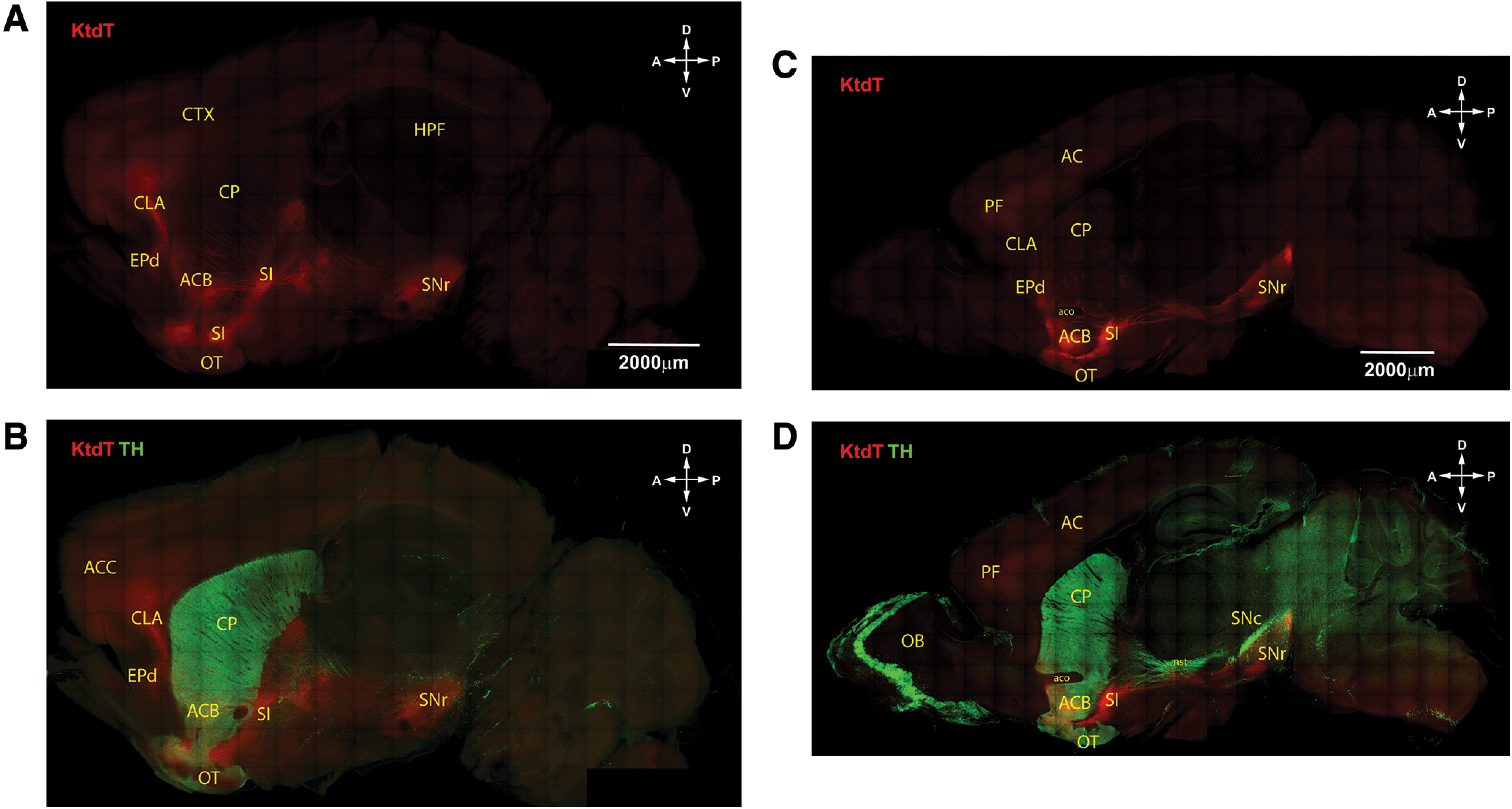
A sagittal brain section (1 mm) of the KtdT mouse showing IHC staining of (***A***) KtdT (red) alone and (***B***) both KtdT (red) and TH (green). See Figure 4 legend. Note that nerve fibers are clearly visible. ***A***, ***B***, This section is ∼2.15 mm lateral to the midline. ***C***, ***D***, This section is ∼1.35 mm lateral to the midline.

Following clearing of brains with CLARITY, some brains were cut into 1-mm sections and IHC was performed. Figure 4*A*,*C* show two horizontal sections at −4.4 and −5.6 mm, respectively, ventral to the bregma. Figure 5*A*,*C* show two sagittal sections 2.15 and 1.35 mm, respectively, lateral to the midline. The thickness of the sections and compilation of the confocal Z-stack images allow visualization of KtdT-containing fibers in the ACB, CP, and SI that run mostly rostral-caudal direction and the dense KtdT fibers connecting the SI and SNr. This is the first observation of these KOR-containing fibers. In addition, the CLA as a contiguous band of brain structure is evident in Figure 4*C*,*D*.

KOR is present in some DA neurons and regulate DA neuron activities in several brain regions, including ACB (Spanagel et al., 1990; Thompson et al., 2000; Margolis et al., 2006; Morales and Margolis, 2017), which have been shown to play important roles in KOR-induced aversion (Chefer et al., 2013). TH, the rate-limiting enzyme responsible for the synthesis of catecholamines (DA, norepinephrine, and epinephrine), was used as a marker for catecholamine-containing neurons.

Figure 4*B*,*D* shows two horizontal sections, and Figure 5*B*,*D* shows two sagittal sections, with TH staining in green and KtdT in red. Areas of co-localization include the ACB, CP, VTA, substantia nigra pars compacta (SNc), and SN pars reticulata (SNr). In contrast, the olfactory bulb and DA cell group A11 express only TH and the CLA contains only KtdT. Because of the thickness of sections and compilation of the confocal Z-stack images, TH and KtdT fibers are readily visible. Note the prominent TH fibers emanating from the VTA and SNc.

### KtdT-containing fibers project from CP/ACB to SI and SNr

**Figure 6.**
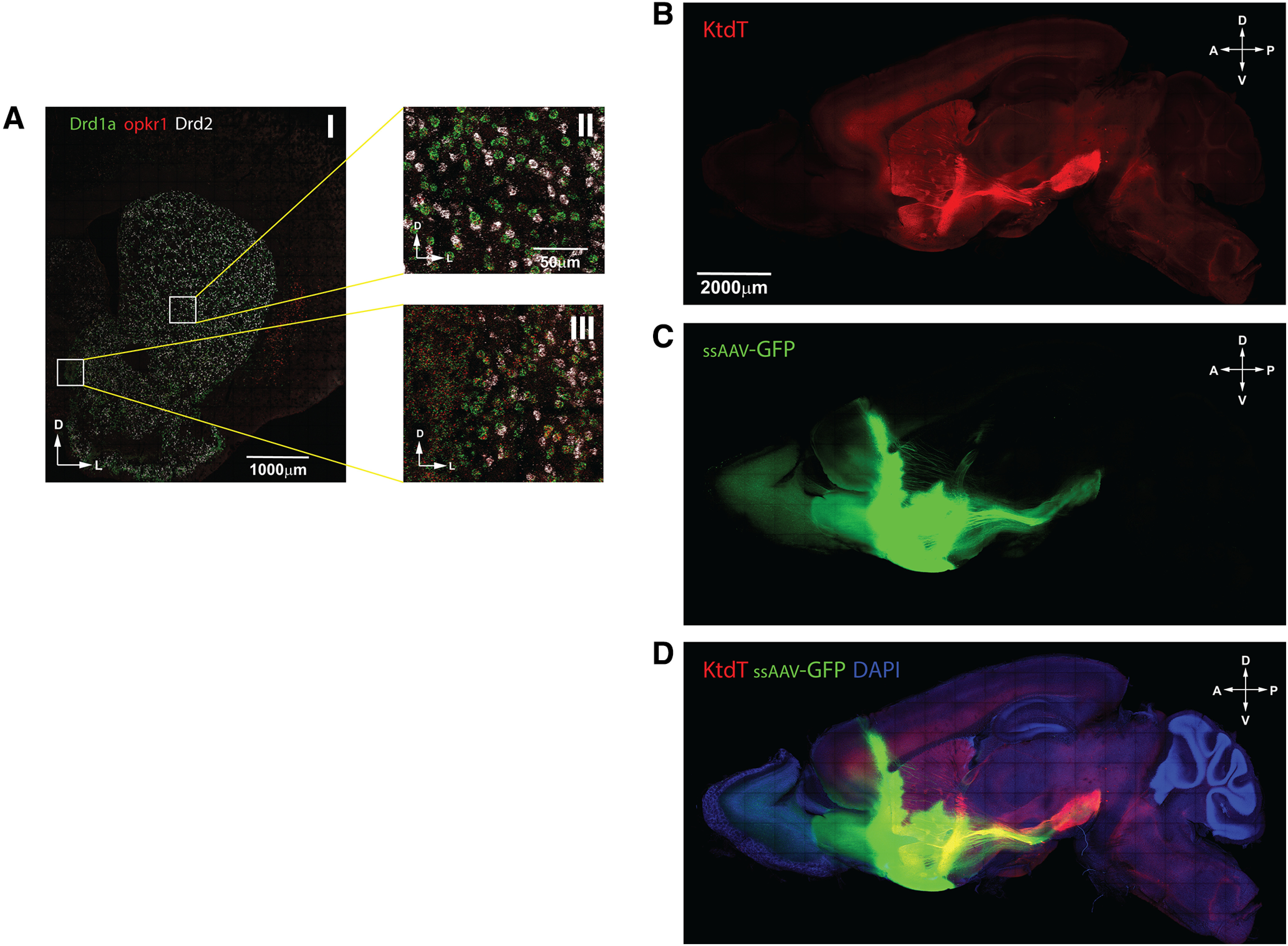
Anterograde tracing shows projection of KtdT-containing neurons from CP and ACB to SI and SNr. ***A***, ISH of KOR mRNA: wild-type C57BL/6N mice were used. ISH was performed with RNAscope on coronal brain sections at regions containing CP and ACB. Experiments were performed on three brains with similar results. Some KOR mRNA (red) is present in D1 (green) or D2 (white) DA receptor-expressing neurons. ***B*–*D***, Anterograde tracing from CP and ACB. Adult KtdT/KtdT mice were injected with scAAV2-GFP tracer (0.2 μl) into the CP and ACB. Three to four weeks after injection, mice were perfused, cleared with CLARITY, processed for KtdT IHC on 1-mm sagittal sections and confocal microscopy as described in Figure 4 legend. ***B***, Red: KtdT. ***C***, Green: scAAV2-GFP. ***D***, Red + green + blue (DAPI). Yellow color indicates overlap of KtdT and scAAV2-GFP. The results indicate projections from CP and ACB to SI and SNr. The experiment was performed three times with similar results.

In Figure 5*A*,*C*, prominent KtdT-expressing fiber bundles are visible in CP, ACB, and SI and between SI and SNr. ISH data showed many KOR mRNA-containing neurons in CP and ACB, most co-localized with the D1-DA receptor (DRD1) or D2-DA receptor (DRD2; Fig. 6*A*), indicating that KOR is expressed in D1-containing or D2-containing neurons. In the ventral striatum, 77% (284/371) of DRD2 cells and 91% (577/634) of DRD1 cells expressed KOR; in the dorsal striatum, 84% (569/677) of DRD2 cells and 94% (674/717) of DRD1 cells expressed KOR. Thus, most medium spiny neurons (MSNs) in the striatum expressed KOR, and it appears that KOR is co-localized with DRD1 to a greater extent than with DRD2. We tested the hypothesis that these KtdT fibers originate from CP and ACB and project to SI and SNr by injecting the anterograde tracer scAAV2-GFP into CP/ACB. As shown in Figure 6*C*, a high level of GFP intensity was detected in the CP, ACB, and SI, which overlapped with KtdT red fluorescence to yield yellow color (Fig. 6*D*). In addition, GFP-containing fibers are observed projecting to the SNr (Fig. 6*C*,*D*). These results indicate that KtdT-containing neurons project from CP/ACB to SI and SNr.

### Co-localization of KtdT and TH at the cellular level in the VTA

**Figure 7.**
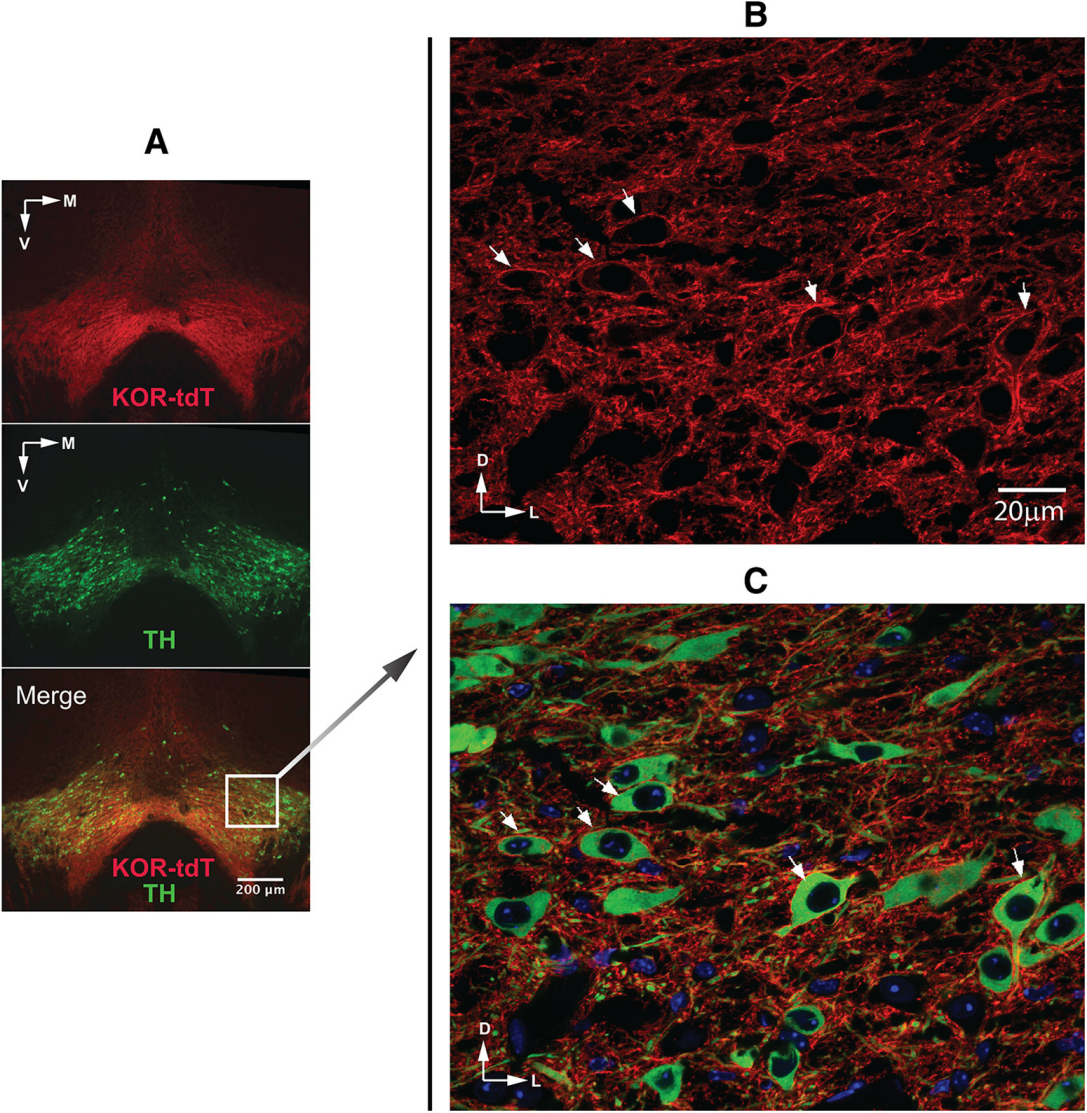
Double IHC staining of KtdT (red) and TH (green) in the VTA. IHC staining for KtdT (red) and TH (green) was performed on coronal sections 30 μm) containing the VTA. ***A***, The three figures show macro views of the VTA region with a 4× objective on a wide-field fluoresce microscope. ***B*** and ***C***, The two figures show KtdT (red) and TH (green) staining acquired with a NikonA1R confocal microscope and a 60× objective, each of which is a MaxIP of three focal planes from a Z-stack. Co-localization of both in some neurons are indicated by arrows. The Z-stacks of confocal images are shown as a video in Movie 3.

We then examined co-localization of KtdT and TH in the VTA at the cellular level. Figure 7*A* shows KtdT staining in red, TH staining in green and merged image, which shows some yellow staining indicating likely co-localization of KtdT and TH in parts of the VTA. In the most ventral and medial portions of the VTA, KtdT staining is not overlapped with TH staining. Confocal microscopy images (Fig. 7*B*) show that KtdT is present mostly on cell membranes and fibers, whereas TH is located most prominently in cytosol. The merged image demonstrates localization of KtdT in some TH-immunoreactive neurons. In Movie 3, a video of Z-stacks of confocal images facilitate identification neurons expressing both TH and KtdT. These results indicate KtdT mouse brain sections allow visualization of co-localization of KtdT and TH at the cellular level.

### U50,488H induced KOR translocation in the VTA

**Figure 8.**
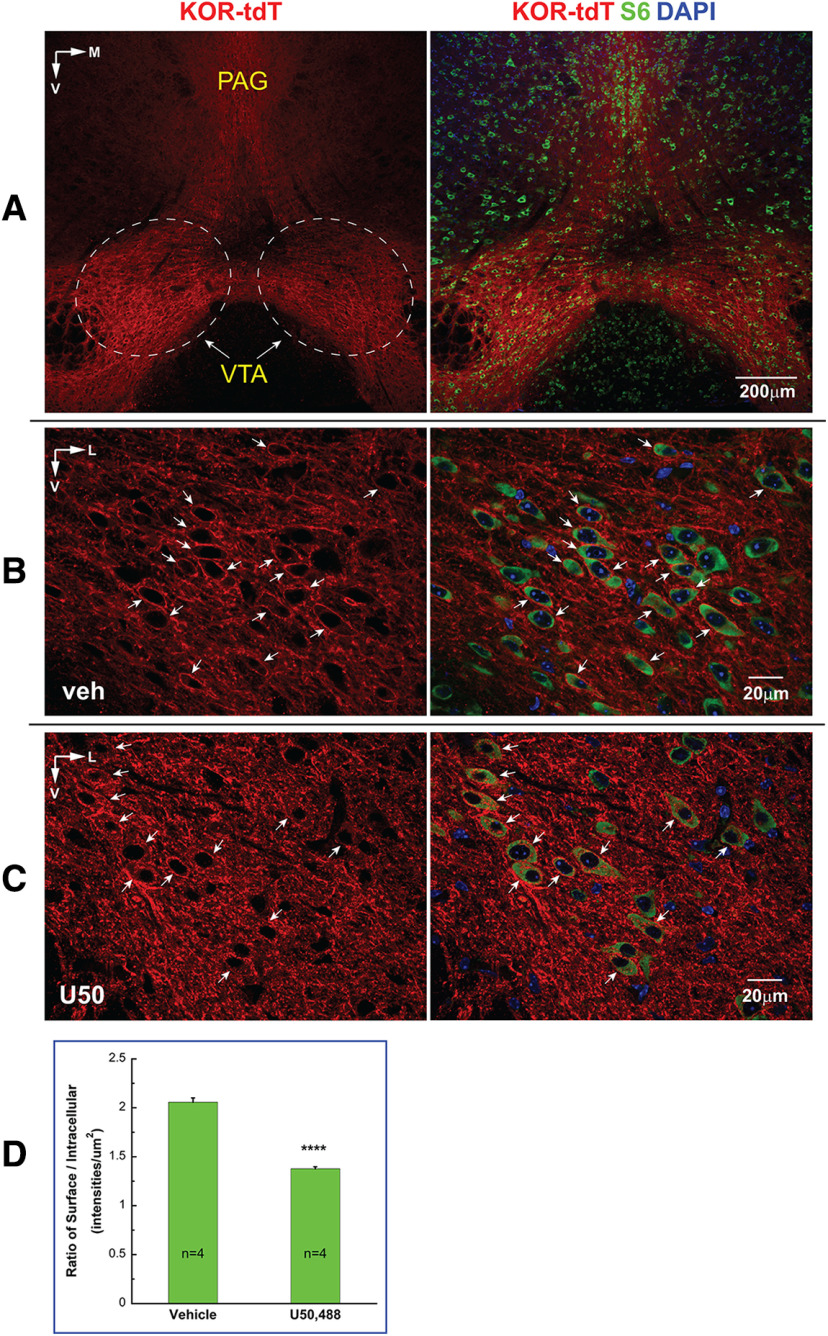
U50,488 induced KOR translocation into cells in the VTA. KtdT/KtdT mice were injected subcutaneously with the agonist U50,488 at 5 mg/kg or vehicle (*n* = 4 each); 30 min later, mice were anesthetized and perfused and coronal sections containing the VTA were processed for IHC for KtdT (red) and ribosomal protein S6 used to define cytosol space (green). ***A***, Macro view of the region containing VTA and PAG. The image was acquired with a 10× objective on confocal microscope. The dotted circles define the area where KtdT neurons were selected for analysis. ***B***, Vehicle group: KtdT in VTA neurons (marked by arrows) are clearly observed as red sharp outlines (most likely in cell membranes). The image is a single focal plane of a Z-stack acquired with a 60× objective on confocal microscope. ***C***, U50,488 group: the image was obtained similarly as in ***B***. Note that U50,488-induced KtdT translocation in KtdT neurons are evidenced by simultaneously exhibiting broken and dotted KtdT surface outlines and punctate staining in cytosol space as contrasted by S6 staining. ***D***, KtdT translocation was quantified with the method described in Extended Data Figure 8-1 and shown as graph. Each value represents mean ± SEM (*n* = 4). Data were analyzed with Student’s *t* test; ****p <* *0.0001.

Figure 8*A* shows a coronal section containing VTA and PAG, both of which express KtdT. KtdT/KtdT mice were treated with vehicle or U50,488 (5 mg/kg, s.c.) and perfused 30 min later. In vehicle-treated mice, KtdT was present on plasma membranes, appearing as a sharp continuous thin line (Fig. 8*B*). Following U50,488H treatment, the KtdT surface sharp line became very dotted along with appearance of punctate staining in cytosol in many VTA neurons (Fig. 8*C*). The intracellular punctate staining is more visible against the green S6 staining (Fig. 8*C*, right panel). These results indicate that agonist treatment causes KtdT translocation from plasma membranes to intracellular space. Quantitation of cell surface and intracellular KtdT revealed a significant reduction in cell surface KtdT 30 min following U50,488H treatment (Fig. 8*D*).

## Discussion

### 3-D KOR distribution in mouse brains

A combination of tagging the KOR with tdT and tissue clearing with CLARITY permitted 3-D imaging of KOR distribution in mouse brains. These are the first 3-D images of distribution of KOR and any G-protein-coupled receptor in mouse brains. One advantage of conjugation of the KOR with tdT is that no IHC is necessary. IHC of cleared brains is very time consuming, often taking weeks, and requires a large amount of antibodies.

The 3-D KOR distribution was identical to that revealed by IHC of KtdT in conventional brain sections, which is similar to that of receptor autoradiography. Technical breakthroughs in brain clearing made it possible to view brain structures in 3-D. Our application of brain clearing technology proves that it is possible to study 3-D distribution of a G-protein-coupled receptor, which will pave ways for more similar studies.

This mouse line also reveals for the first time a major KOR projection from ACB/CP to SI and SNr. Moreover, the KtdT mice allow visualization of KOR at the cellular level, for example, detection of co-localization of KOR with TH and receptor translocation.

The confocal microscope used in this study was not optimized for imaging cleared whole brain due to its nature of being a shared resource, which gave rise to imperfections visible as stitch shadings in the 3-D whole-brain image as well as in the 1-mm section images. We are aware that the image quality could be improved with better optical hardware such as a light sheet microscope or a confocal microscope optimized for cleared whole brain. However, we chose to use the confocal microscope because it was readily accessible, an important consideration given the workload of the experiment. In addition, we noticed low signal/noise of the KtdT in brain, which also contributed to stitch shadings and noises in the image because much higher laser power required exacerbated photobleaching. Increasing sampling to near Nyquist parameters did not improve shading significantly, but markedly increased image acquisition time. An extensive discussion of optical refinements is beyond the scope of this report.

### CP/ACB projection to SNr expresses KOR

The high level of KtdT observed in SN is largely confined to the SNr, whereas most of DA neurons are localized in SNc, with only a small fraction of co-localization. A major KtdT-expressing neuronal fiber bundle between CP/ACB and SNr was observed. The observation of highly visible KOR-containing fibers may be due to the interaction of KOR with GEC1, a microtubule-associated protein, which facilitates transport of KOR along the neuronal fibers (Chen et al., 2006). Our anterograde tracing results demonstrated that KtdT-containing fibers project from CP/ACB to SNr. The pathways projecting from dorsomedial striatum or ACB to SNr were demonstrated previously (Soares-Cunha et al., 2016; Cheng et al., 2017); our result is the first to reveal that this pathway expresses KOR. SNr receives projections from neurons in the striatum via direct and indirect pathways (Waxman, 2017). The direct pathway originates from DRD1/dynorphin MSNs and exerts inhibitory effects on SNr neurons. The indirect pathway, initiating from DRD2/enkephalin MSNs, via the globus pallidus and subthalamic nucleus, exerts excitatory actions on SNr neurons. By ISH, we found that some KOR mRNA in the CP/ACB was present in DRD1-expressing or DRD2-expressing neurons. Thus, it is likely that the KOR is present on both direct and indirect pathways. Cheng et al. (2017) reported that both D1-MSNs and D2-MSNs in dorsomedial striatum are involved in alcohol consumption and KOR on these neurons may regulate their activities.

### KtdT mice enabled visualization of KtdT at the cellular level

Co-localization of KtdT with TH immunoreactivities was observed within many brain regions, particularly in limbic structures and the basal ganglia. With confocal microscopy co-localization of KtdT and TH was visualized at the cellular level (Fig. 7*B*). KtdT is present on plasma membranes of neuronal cell bodies and in nerve fibers, whereas TH is in cytosol. A video of Z-stack images shown in Movie 3 facilitates identification of co-localization.

#### KOR translocation following U50,488H administration

U50,488H caused KtdT movement into intracellular space in the VTA, which was visualized as markedly reduced cell surface KtdT and increased punctate KtdT in cytosol.

### Functional neuroanatomy of the KOR

In the following sections, we briefly discuss the KOR in the brain regions and pathways known to have roles in KOR-mediated behaviors, including aversion, anxiety, addiction, pain processing, and neuroendocrine functions. More importantly, we highlight several brain regions that have high KOR levels, but functions of these KORs have not been established. Possible roles of these KORs in KOR-mediated behaviors are discussed.

### KOR in brain regions and pathways with identified functions for KOR

#### KOR in the areas involved in mood, reward, motivation, and addiction

KOR was observed in the VTA, ACB, PF, anterior cingulate cortex, amygdala nuclei, bed nucleus of stria terminalis (BST), and raphe nucleus, with ACB shell exhibiting particularly intense signal. The VTA neurons project to the ACB, PF and amygdala. KOR in VTA ACB pathway plays a critical role in aversion induced by KOR agonists and following drug abuse (Van't Veer et al., 2013; Morales and Margolis, 2017). Activation of KOR in the ACB reduces DA release and increases DA reuptake (Spanagel et al., 1990; Thompson et al., 2000), which play important roles in KOR-induced aversion (Chefer et al., 2013). Repeated stress induces dynorphin-dependent KOR activation in the BLA, ACB, dorsal raphe, and HIP, which is responsible for the aversive component of stress (Land et al., 2008). KOR activation in the BLA, central amygdala (CEA), and BST appear to be associated with anxiety, stress, drug and alcohol abuse, and aversion associated with pain (Bruchas et al., 2009; Knoll et al., 2011; Kallupi et al., 2013; Navratilova et al., 2019). Activation of the KOR in serotoninergic neurons in the dorsal raphe nucleus–ACB pathway is responsible for KOR agonist-induced and stress-induced aversion and caused stress-induced drug seeking (Land et al., 2009).

#### KOR in pain pathway

Moderate levels of KtdT are present in the PAG, parabrachial nucleus, some nuclei in thalamus, and primary and secondary somatosensory cortices, areas known to be involved in pain transmission and modulation. The presence of KtdT in the PAG is consistent with analgesic effects of KOR agonists (von Voigtlander et al., 1983). The spinal cord and dorsal root ganglia also express modest levels of KOR, which is outside of the scope of the current study.

#### KOR in hypothalamus

KtdT is widely distributed in the hypothalamus, indicating roles of KOR in neuroendocrine functions. KOR is involved in regulation of release of hormones, such as antidiuretic hormone, luteinizing hormone, prolactin, and oxytocin (Leander et al., 1985; Leadem and Yagenova, 1987; Leng et al., 1997; Butelman et al., 1999), likely through KOR in the hypothalamus.

### Brain regions that have high to moderate KOR expression, but functional roles of KOR are unclear

#### CLA and EP

The CLA and EPd have very high levels of KOR. The CLA, a long thin band-like subcortical gray matter in the forebrain, has extensive reciprocal connections with the cortex and, to lesser extents, with subcortical regions (Mathur, 2014; Wang et al., 2017). It was hypothesized to be the seat of consciousness (Crick and Koch, 2005). The EP sends extensive projections to most basal forebrain areas including the piriform, entorhinal, insular, and orbital cortices, and all cortical amygdala areas (Behan and Haberly, 1999). The EP was suggested to be an area of convergence for sensory and affect-related information and involved in aspects of the storage, consolidation, and retrieval of emotional memories (de Curtis and Paré, 2004). KOR agonists cause hallucinations and dysphoria (Pfeiffer et al., 1986; Roth et al., 2002). The KOR in the CLA and EP may be involved in KOR-mediated cognitive and perceptual effects.

PVT and RE, substructures of the midline thalamic nuclear group, show moderate levels of KtdT. PVT receives innervation from the prelimbic cortex, raphe, PAG, and hypothalamus and sends projections to the ACB, amygdala, and LS. Thus the PVT relays stress signals from the former to the latter (Hsu and Price, 2009) as part of a circuit that manages stress and possibly causes stress-related psychopathologies. The RE receives widespread projections from limbic and limbic-associated structures (McKenna and Vertes, 2004) and sends efferent to the HIP, medial PF, and entorhinal cortex (Wouterlood et al., 1990; Vertes, 2006). These pathways are important in attentiveness and resilience to stress, among other functions. Little is known about functional significance of KOR in these nuclei.

#### LHb

LHb exhibited a moderate level of KtdT. LHb received inputs from forebrain structures, most importantly PF, SI, globus pallidus, and lateral hypothalamus. LHb sends projections to DA neurons in the VTA and retromedial tegmental nucleus and serotonin neurons in raphe nuclei (for review, see Boulos et al., 2017; Zhou et al., 2018). Thus, LHb connects cognitive to emotional and sensory processing (Boulos et al., 2017). In animals, inhibition of LHb activity is associated with depression-like behaviors and stimulation of LHb shows antidepressant-like activity (Yang et al., 2018). The KOR in this brain region may play a role in depression-like behavior caused by KOR activation.

### Limitations of the KtdT mouse line

There are quantitative differences in KOR density in some brain regions. For example, SNr and PAG showed higher KtdT levels than expected from [^3^H]U69,593 autoradiography in wild type. In the brain, KtdT/KtdT had higher levels of KOR protein and mRNA and U50,488H showed higher potency in KtdT/KtdT mice than the wild-type mice. Conjugation of the KOR to tdT may affect stability and transport of the receptor.

As seen in the VTA (Fig. 8*B*,*C*), most of KtdT is present on membranes and fibers, and it is difficult to visualize KtdT-expressing neuronal cell bodies. Counterstaining of cytosol helps, but it is often necessary to go along the Z planes of confocal images to be certain (Movie 3; Extended Data Fig. 8-1).

## Synthesis

Reviewing Editor: Kirill Martemyanov, The Scripps Research Institute Decisions are customarily a result of the Reviewing Editor and the peer reviewers coming together and discussing their recommendations until a consensus is reached. When revisions are invited, a fact-based synthesis statement explaining their decision and outlining what is needed to prepare a revision will be listed below. The following reviewer(s) agreed to reveal their identity: Bronwyn Kivell. Note: If this manuscript was transferred from JNeurosci and a decision was made to accept the manuscript without peer review, a brief statement to this effect will instead be what is listed below.

Upon discussion the reviewers agreed on the significance of the work and the technical quality for the most part and as such support the publication. Reviewers also thought that revisions cold significantly improve the manuscript. Reviewers made a number of technical comments addressing which would be necessary for the manuscript to be published. It was also felt that attention to the application of statistical methods would be required. Please refer to specific reviewers’ comments below.

Reviewer #1

Localisation of KOR in tissue has been plagued by poor quality antibodies, particularly with antibodies lacking specificity. While RNA scope has provided detailed localisation of message, there is gap in our ability to look at protein localisation in detail within brain slices, particularly withing the intact brain. Recent advances in lightsheet microscopy and tissue clearing procedures has made whole-brain imaging widespread and this KOR-TdTomato mouse will provide a valuable research tool to study KOR localisation and expression in a variety of applications.

This paper is likely to be of significant interest to a wide audience and all scientists interested in the function and localisation of KOR. In fact, it is hard to believe that this is the first to present 3-D localisation of a GPCR in the intact brain, but this seems to be the case, which is of particular interest.

The team has created an important research tool for scientists studying KOR and addresses a large gap regarding the lack of selective antibodies to identify KOR in tissue sections.

Suggested Revisions:

1) define all abbreviations in text 1st use.

2) It is not clear from the methods whether behavioral or

image quantification analysis was performed by an observer blinded to treatment groups (?) This needs to be clarified.

3) Results: pg 16: “ratio of 3.6:1 in

the wildtype vs. KtdT/KtdT mouse brain (Fig. 1D)” should read ...ratio of 1:3.6 ...... respectively.

4)Figure 2: note that standard confocal microscopy was used to construct 3-D images of whole cleared mouse brains. While the images are ok, there are significant artifacts giving poor image quality, the visible dark squares in stitched images are a result of not using Niquist sampling. Also, standard confocal microscopy has limited laser penetration even in cleared tissue. I suspect confocal settings were used that compensated and adjusted for this? but this is not clear from the methods, and should be stated. This is important when comparing staining intensities in tissues located at different tissue depths, even in cleared tissue. I am wondering, given the importance of this why lightsheet microscopy was not used? At a minimum, discussion of standard confocal microscopy limitations should be discussed regarding imaging whole brain.

5) Quantification of data in fig 8. lacks detail and without added detail on how this was performed is not particularly convincing. N=2 mice were indicated for Nor-BNI experiments but number of mice used for other treatments have not been indicated. Given that this method of using FST to internalize KOR is not an established protocol to screen for KOR function, further details are needed. For example how were the KOR cells used for analysis selected? It would help to also include a low magnification picture to see KOR distribution in a larger area. Statistical analysis appears to have been performed on number of cells, shoudn’t the ‘internalisation score’ be averaged per mouse and this compared between FST and control mice to give true statistical validation?

Co-labeling with an early endosome marker would confirm/validate an increase in KOR internalization following FST exposure. ALternatively, evaluate whether KOR is functional in this mouse model via electrophysiological techniques.

Reviewer #2

In this manuscript, Chen et al characterize a novel knock-in mouse line expressing a kappa opioid receptor-tdTomato fusion protein. To demonstrate the validity of the mouse line, the authors perform CLARITY, highlighting the utility of this line for use without antibodies, and IHC, comparing the expression of td-Tomato to [3H]U69,593 binding. Below are a few concerns, which if addressed, would bolster the narrative of the manuscript and aid in my recommendation for publication. While the overall report of this tool is useful, I highly recommend the authors address important issues regarding clarity in the manuscript, and revision of many of the figures, which appear low quality and hard for the reader to appreciate fluorescence intensities in certain brain regions, as well as some investigation of baseline behavioral and physiological differences between this knock-in animal compared to WTs.

1. The authors show higher mRNA and [3H]U69,593 binding in KOR-tdTomato mice, suggesting over-expression of functional KOR, corroborated by the heightened sensitivity to U50 at preventing scratching, compared to WT. While the authors show that “functional KOR expression” is similar via autoradiography and IHC (Fig. 3A) and address this as a limitation in the discussion, I recommend that the authors perform qPCR to analyze mRNA levels and [3H]U69,593 binding to quantify binding in one region with high, moderate and low KOR expression each.

2. The authors would benefit greatly from performing more behavioral validation experiments; for eg., they do not provide behavioral data with respect to the forced swim tests performed to determine KOR translocation. If KOR-tdTomato mice help demonstrate that KOR is internalized upon forced-swim stress, blocked by NorBNI, does the behavior data corroborate this? Furthermore, what does immobility look like in the KOR-tdTomato mice compared to WT and their sensitivity to NorBNI?

3. The readers will benefit from defining the fluorescent markers in green and red in Fig. 4B,D and 5B,D.

4. What is the scale in the magnified image in 6A? On a similar note, the readers will benefit if the authors kept the measurements of the scale consistent (i.e., mm vs. um) on the images.

5. The readers will benefit from a compass on the images.

6. In Fig. 3B, including magnified images of td-Tomato expression, especially in low to moderate KOR-expressing regions would benefit the narrative of the manuscript greatly.

7. The article is filled with typographic and grammatical inconsistencies. The authors (and ultimately, readers) would greatly benefit from editing the manuscript more thoroughly before it is published.

8. mRNA and [3H]U69,593 binding was higher in knock-in mice, suggesting over-expression of functional KOR, as well as increased sensitivity to anti-scratch action of U50 at increasing doses compared to WTs. Does this change in KOR sensitivity/expression have behavioral and physiological consequences (i.e., changes in hormone levels regulated by KOR neurons?) Do animals display greater stress sensitivity compared to WTs in line with KOR-mediated actions on certain behaviors?

9. Merge 3-D image and IHC results section with the “Two approaches...” section. There is no need to separate out sections which do not refer to figures directly.

10. Images throughout manuscript appear very pixelated-please provide the high res versions.

11. Please provide a graphic in panels in Fig 2C like in 2A so we can see where in the 3-D image you picked for the 1mm image close-ups.

12. It would be better to show a close-up panel of PAG because it is really hard to see fluorescence changes from the image presented here with low objective magnification.

13. Where is Figure 3C?

14. Can you reference individual Figure panels when you refer to them in the text? It would be helpful to innumerate each picture with (i, ii, etc) or just keep the A-B-C nomenclature within each figure to individual panel designation.

15. Please abbreviate the in-text brain areas consistent with labeling in figure (for example in the text, mention abbreviation after full name of medial preoptic area (MPO) and all other areas mentioned.

16. It is confusing why the authors mention 4B and 4D and then jump to Figs. 5B and 5D. Please keep the order of figures in line with their reference in the text.

17. It would be beneficial for other researches reading your manuscript if you could quantify degree of co-localization of DRD1 and DRD2 with oprk1 in the ventral and dorsal striatum.

Methods-

Please correct grammatical error in the following sentence:

"To gain tissue transparency, while maintain maintaining the integrity of the brain structure and reduce reducing the time needed, we adapted the electrophoretic tissue clearing method described by (Kim et al., 2015).

## Author Response

We appreciate the reviewers’ constructive comments. The following are our complete responses (in blue). The reviewers’ comments are also included below.

Responses to Reviewer #1

1) define all abbreviations in text 1st use.

This has been done per reviewer’s suggestion.

However, we retain the abbreviations for brain regions and nuclei in Fig. 3 legend, which are necessary for deciphering the labels in the images shown in figures.

2) It is not clear from the methods whether behavioral or image quantification analysis was performed by an observer blinded to treatment groups (?) This needs to be clarified.

Image quantitation analyses were done by an observer blinded to the groups. The following has been added on page 13:

"Image quantitation analyses were done by an observer blinded to the treatment groups.”

Counting of scratching bouts was done live, so it was not blind. Locomotor activities were recorded with a Locomotor Activity System (Omnitech Electronics Inc., Columbus, OH), so there was no involvement of any researcher in recording activities.

3) Results: pg 16: “ratio of 3.6:1 in

the wildtype vs. KtdT/KtdT mouse brain (Fig. 1D)” should read ...ratio of 1:3.6 ...... respectively.

This has been corrected in page 14.

4) Figure 2: note that standard confocal microscopy was used to construct 3-D images of whole cleared mouse brains. While the images are ok, there are significant artifacts giving poor image quality, the visible dark squares in stitched images are a result of not using Niquist sampling. Also, standard confocal microscopy has limited laser penetration even in cleared tissue. I suspect confocal settings were used that compensated and adjusted for this? but this is not clear from the methods, and should be stated. This is important when comparing staining intensities in tissues located at different tissue depths, even in cleared tissue. I am wondering, given the importance of this why lightsheet microscopy was not used? At a minimum, discussion of standard confocal microscopy limitations should be discussed regarding imaging whole brain.

We agree with most of reviewer’s comments, if not all. Indeed, light sheet microscopy is by far a better tool than confocal microscopy for cleared whole brain imaging. However, we do not have easy access to light sheet settings, because of outsourcing expenses and waiting time. We settled with confocal microscopy because it was readily accessible to us, which was important given the workload of the experiment. In addition, we noticed low signal/noise of the KtdT in brain, which also contributed to stitch-shadings and noises in the image because much higher laser power required exacerbated photobleaching. Increasing sampling to near Nyquist parameters did not improve shading significantly (it went to 35h from 12h on acquisition time with 10X obj.). We are not certain how much better light sheet can do in our case, which may take multi-rounds for trial and error. But we did try TissueCyte of TissueVision once, a powerful tool for large tissue 3D imaging. To our disappointment, it came up with a 3D whole brain image showing low KtdT specific signals. They cited low S/N value as a major obstacle for any success. While we are aware of the challenges and imperfections, we truly appreciate that the images are given an OK for their quality by the reviewer.

The parameters used for Z-depth compensation have been added to page 11 as follows:

"Z-correction (to compensate signal reduction along Z-depth) from Z-depth 0 to 3.5 mm: laser power was set 10 to 20 for Ch3, 20 to 30 for Ch2 correspondingly; PMT HV was set 20 to 40 for Ch3, 20 to 40 for Ch2 correspondingly.”

The following paragraph has been added to page 21 in Discussion to acknowledge the cause of image imperfection:

"The confocal microscope used in this study was not optimized for imaging cleared whole brain due to its nature of being a shared resource, which gave rise to imperfections visible as stitch-shadings in the 3D whole brain image as well as in the 1-mm section images. We are aware that the image quality could be improved with better optical hardware such as a Light Sheet microscope or a confocal microscope optimized for cleared whole brain. However, we chose to use the confocal microscope because it was readily accessible, an important consideration given the workload of the experiment. In addition, we noticed low signal/noise of the KtdT in brain, which also contributed to stitch-shadings and noises in the image because much higher laser power required exacerbated photobleaching. Increasing sampling to near Nyquist parameters did not improve shading significantly, but markedly increased image acquisition time. An extensive discussion of optical refinements is beyond the scope of this report.”

5) Quantification of data in fig 8. lacks detail and without added detail on how this was performed is not particularly convincing. N=2 mice were indicated for Nor-BNI experiments but number of mice used for other treatments have not been indicated. Given that this method of using FST to internalize KOR is not an established protocol to screen for KOR function, further details are needed. For example how were the KOR cells used for analysis selected? It would help to also include a low magnification picture to see KOR distribution in a larger area. Statistical analysis appears to have been performed on number of cells, shoudn’t the ‘internalisation score’ be averaged per mouse and this compared between FST and control mice to give true statistical validation?

Co-labeling with an early endosome marker would confirm/validate an increase in KOR internalization following FST exposure. ALternatively, evaluate whether KOR is functional in this mouse model via electrophysiological techniques.

We agree with the reviewer that the correlation between FST and KOR translocation is yet to be established. To stay focus on our topic, we now present data on KOR translocation induced by the prototypic KOR agonist U50,488H. The results turned out to be quite convincing to us. We therefore revised the Fig. 8 with the new images, including a macro view of VTA region (We suggest that you zoom in the images for better details). Nor-BNI was known to block U50,488H effect in previous studies, therefore it was not tested in this experiment. We also think that an endosome marker is not needed because the typical pattern of KtdT translocation is obvious. We thus use the term translocation instead of internalization. The highly anisotropic nature of KtdT neurons was observed and thus a major factor taken into consideration to develop the quantification method. The method has been detailed in the revised Fig. 8-1 and its legend. Statistical analysis has been performed as suggested and shown in Fig. 8D. Accordingly, all changes have been made to the texts and figure legends. See p. 13 for the methods, p. 19-20 for the results, p. 23 for discussion, Figure 8 and Figure 8 legend. We hope that the reviewers find these revisions satisfactory.

Responses to Reviewer #2

1. The authors show higher mRNA and [3H]U69,593 binding in KOR-tdTomato mice, suggesting over-expression of functional KOR, corroborated by the heightened sensitivity to U50 at preventing scratching, compared to WT. While the authors show that “functional KOR expression” is similar via autoradiography and IHC (Fig. 3A) and address this as a limitation in the discussion, I recommend that the authors perform qPCR to analyze mRNA levels and [3H]U69,593 binding to quantify binding in one region with high, moderate and low KOR expression each.

In comparison with WT mice, KtdT in homozygous mice demonstrated higher mRNA level (∼3.6 times) and much higher protein level (>10 times) in terms of the whole brains. This indicated that conjugation of the KOR to tdT may affect stability and/or transport of the receptor at both mRNA and protein levels. Because the distribution of KtdT immunoreactivity is consistent with that of [3H]U69,593 autoradiography in the wildtype mouse brain, the increase of KtdT receptor levels is likely to occur in most (if not all) KOR-expressing regions in an proportional and brain-region independent manner. Furthermore, our studies mostly focused on qualitative anatomical characterization of KtdT. Thus, we respectfully disagree with the reviewer’s suggestion to perform region-specific studies on KtdT expression.

There are also two technical reasons why we decided against doing the [3H]U69,593 binding experiments at this time. We believe that without KtdT protein data, KtdT mRNA data are not as meaningful.

• KOR level in the wildtype mouse brain is very low. We had to use a large amount of brain membranes (∼1.8 mg protein/tube) for [3H]U69,593 (∼4.5 nM) binding experiments and even at this high protein concentration, the specific binding was only ∼200 dpm/tube in the WT mice (see Fig. 1C). In fact, the high amount of brain membranes used almost clogged the GF/B filters. For [3H]U69,593 binding to brain regions, we would have to pool tissues from a large number of mice, particularly low KOR-expressing regions in the WT mice. We thus opted not to do it.

• Alternatively, autoradiography of [3H]U69,593 binding to KOR in sections of KtdT mouse brains may be performed. It is known that autoradiography of [3H]-labeled ligand takes a long time. We thus decided against doing this experiment at this time.

2. The authors would benefit greatly from performing more behavioral validation experiments; for eg., they do not provide behavioral data with respect to the forced swim tests performed to determine KOR translocation. If KOR-tdTomato mice help demonstrate that KOR is internalized upon forced-swim stress, blocked by NorBNI, does the behavior data corroborate this? Furthermore, what does immobility look like in the KOR-tdTomato mice compared to WT and their sensitivity to NorBNI?

We have replaced FST-induced KOR translocation with U50,488H-induced translocation. We agree that it is interesting to compare WT and KtdT mice in the FST test following KOR antagonist treatment. However, we think it is outside of the main focus of the manuscript, which is neuroanatomical characterization. See our responses to Reviewer 1 point 5.

3. The readers will benefit from defining the fluorescent markers in green and red in Fig. 4B,D and 5B,D.

The markers have been added to the figures.

4. What is the scale in the magnified image in 6A? On a similar note, the readers will benefit if the authors kept the measurements of the scale consistent (i.e., mm vs. um) on the images.

The scale bars (in μm) have been added to Fig. 6A and all the other figures.

5. The readers will benefit from a compass on the images.

The compass has been added to Fig. 2. The other images are conventional coronal, sagittal and horizontal sections; therefore, we believe that it is not necessary to add a compass.

6. In Fig. 3B, including magnified images of td-Tomato expression, especially in low to moderate KOR-expressing regions would benefit the narrative of the manuscript greatly.

Three higher resolution images have been added as Fig. 3C, which represent regions with relative KtdT intensities from high to low.

7. The article is filled with typographic and grammatical inconsistencies. The authors (and ultimately, readers) would greatly benefit from editing the manuscript more thoroughly before it is published.

We have proofread the manuscript carefully.

8. mRNA and [3H]U69,593 binding was higher in knock-in mice, suggesting over-expression of functional KOR, as well as increased sensitivity to anti-scratch action of U50 at increasing doses compared to WTs. Does this change in KOR sensitivity/expression have behavioral and physiological consequences (i.e., changes in hormone levels regulated by KOR neurons?) Do animals display greater stress sensitivity compared to WTs in line with KOR-mediated actions on certain behaviors?

These are intriguing questions. We do not know the answers yet. The huge increase in KtdT expression may lead to unexpected behavioral and physiological consequences in KtdT mice. On the other hand, as the serendipitous enrichment of KtdT greatly facilitated KOR detection, we focused on anatomical characterization of KtdT in current studies and accomplished the 3-D images without IHC staining. The KtdT mice may be used to address these questions, however we believe they are outside of the scope of the current study.

9. Merge 3-D image and IHC results section with the “Two approaches...” section. There is no need to separate out sections which do not refer to figures directly.

The sections have been merged as two sub-sections under the section: Two approaches for examination of distribution of KtdT in the mouse brain on pages 14-15.

10. Images throughout manuscript appear very pixelated-please provide the high res versions.

All images have been scaled to >= 300 dpi from original large image files and embedded in eps files (we resubmit all figures in eps format in the revision).

11. Please provide a graphic in panels in Fig 2C like in 2A so we can see where in the 3-D image you picked for the 1mm image close-ups.

A horizontal view of whole brain image has been added to Fig 2C to assist with visualization of the close-ups.

12. It would be better to show a close-up panel of PAG because it is really hard to see fluorescence changes from the image presented here with low objective magnification.

A new image including the PAG and VTA has been added to Fig 8A which can be used to draw a comparison between the two regions. It was taken with 10X obj., not a very high resolution, but you can zoom in to view neurons in both regions. Hope you find the images useful.

13. Where is Figure 3C?

Sorry, it was a typo and it should be 3B. This has been corrected in page 16. A new figure has been added as Fig. 3C (see Reviewer #2 point 6).

14. Can you reference individual Figure panels when you refer to them in the text? It would be helpful to innumerate each picture with (i, ii, etc) or just keep the A-B-C nomenclature within each figure to individual panel designation.

This has been done per reviewer’s suggestion.

15. Please abbreviate the in-text brain areas consistent with labeling in figure (for example in the text, mention abbreviation after full name of medial preoptic area (MPO) and all other areas mentioned.

The changes have been made per reviewer’s suggestion.

16. It is confusing why the authors mention 4B and 4D and then jump to Figs. 5B and 5D. Please keep the order of figures in line with their reference in the text.

4B, 4D, 5B and 5D have both KtdT and TH staining, whereas 4A, 4C, 5A and 5C have only KtdT staining. 4B and 4D are corresponding images of 4A and 4B, respectively and 5B and 5D are counterparts of 5A and 5D, respectively. We think it is more logical to keep the way it was.

17. It would be beneficial for other researches reading your manuscript if you could quantify degree of co-localization of DRD1 and DRD2 with oprk1 in the ventral and dorsal striatum.

Quantitation was carried out on coronal brain sections and the results have been added in page 18 as follows.

"In the ventral striatum, 77% (284/371) of DRD2 cells and 91% (577/634) of DRD1 cells expressed KOR; in the dorsal striatum, 84% (569/677) of DRD2 cells and 94% (674/717) of DRD1 cells expressed KOR. Thus, most MSNs in the striatum expressed KOR and it appears that KOR is co-localized with DRD1 to a greater extent than with DRD2.”

Methods-

Please correct grammatical error in the following sentence:

"To gain tissue transparency, while maintain maintaining the integrity of the brain structure and reduce reducing the time needed, we adapted the electrophoretic tissue clearing method described by (Kim et al., 2015).

This has been corrected.
